# What Affects Perceived Trustworthiness of Online Medical Information and Subsequent Treatment Decision Making? Randomized Trials on the Role of Uncertainty and Institutional Cues

**DOI:** 10.1177/23814683241226660

**Published:** 2024-02-15

**Authors:** Gabriel Recchia, Karin S. Moser, Alexandra L.J. Freeman

**Affiliations:** Winton Centre for Risk & Evidence Communication, Department of Pure Maths and Mathematical Statistics, University of Cambridge, Cambridge, UK; UniDistance Suisse, Faculty of Psychology, Brig, Switzerland; Winton Centre for Risk & Evidence Communication, Department of Pure Maths and Mathematical Statistics, University of Cambridge, Cambridge, UK

**Keywords:** trust, algorithms, medical decision making, institutional cues, uncertainty, psychological distance

## Abstract

**Background.** Online, algorithmically driven prognostic tools are increasingly important in medical decision making. Institutions developing such tools need to be able to communicate the precision and accuracy of the information in a trustworthy manner, and so many attempt to communicate uncertainties but also use institutional logos to underscore their trustworthiness. Bringing together theories on trust, uncertainty, and psychological distance in a novel way, we tested whether and how the communication of uncertainty and the presence of institutional logos together affected trust in medical information, the prognostic tool itself, and treatment decisions. **Methods.** A pilot and 2 online experiments in which UK (experiment 1) and worldwide (experiment 2) participants (N_total_ = 4,724) were randomized to 1 of 12 arms in a 3 (uncertainty cue) × 4 (institutional cue) between-subjects design. The stimulus was based on an existing medical prognostic tool. **Results.** Institutional trust was consistently associated with trust in the prognostic tool itself, while uncertainty information had no consistent effect. Institutional trust predicted the amount of weight participants reported placing on institutional endorsements in their decision making and the likelihood of switching from passive to active treatment in a hypothetical scenario. There was also a significant effect of psychological distance to (perceived hypotheticality of) the scenario. **Conclusions/Implications.** These results underline the importance of institutions demonstrating trustworthiness and building trust with their users. They also suggest that users tend to be insensitive to communications of uncertainty and that communicators may need to be highly explicit when attempting to warn of low precision or quality of evidence. The effect of the perceived hypotheticality of the scenario underscores the importance of realistic decision-making scenarios for studies and the role of familiarity with the decision dilemma generally.

**Highlights:**

- In a world where information for medical decision making is increasingly going to be provided through digital, online tools, institutions providing such tools need guidance on how best to communicate about their trustworthiness and precision.
- We find that people are fairly insensitive to cues designed to communicate uncertainty around the outputs of such tools. Even putting “ATTENTION” in bold font or explicitly pointing out the weaknesses in the data did not appear to affect people’s decision making using the tool’s outputs. Institutions should take note, and further work is required to determine how best to communicate uncertainty in a way that elicits appropriate caution in lay users.
- People were much more sensitive to institutional logos associated with the outputs. Generalized institutional trust (rather than trust in the specific institution whose logo was shown) was associated with how trustworthy, accurate, and reliable the tool, its algorithm, and the numbers it produced were perceived to be. This underscores the role of societal trust in institutions at large.
- Finally, as a note to researchers, we found a significant effect of how hypothetical or believable participants felt the experimental scenario was. This is a variable that seems rarely controlled for in studies and yet played as much of a role as some of our variables of interest, so we suggest that it is measured in future experiments.

## Introduction

Online tools that provide personalized information about the potential outcomes of different choices already play an important role in the decision making of clinicians and patients, such as Predict:Breast Cancer,^1^ Predict:Prostate,^2^ CanRisk,^3^ FRAX,^4^ HeartAge,^5^ and QRisk,^6^ among others. In shared decision-making contexts, clinicians and patients use the outputs of such tools to inform their decisions on which treatments, if any, to pursue.

For organizations that develop such tools, to present information in a way that is evidence based, trustworthy, and easy to understand for both professionals and laypeople involves communicating uncertainty about risks, and data quality as well as communicating who stands behind the quality and reliability of the tool and the underlying data and algorithms.

While previous research has investigated determinants of online trust and of communicating uncertainty of algorithmically derived data, there is a lack of research systematically investigating how institutional and uncertainty cues may affect trust in online tools and affect the subsequent decision making of users. We address this by bringing together relevant theoretical frameworks: a recently developed framework for communicating epistemic uncertainty,^7^ trust transference theory,^8^ and Cazier’s^9^ extension of Mayer et al.’s venerable integrative model of organizational trust.^10^ Moreover, we also include the hypotheticality^11^ of the treatment scenario. Psychological distance is rarely included in experimental research using hypothetical scenarios, but we would argue it is essential in assessing and explaining the transferability of experimental results into practice. To further increase the generalizability and relevance of our research, we are using a decision scenario that is based on a commonly and internationally used medical prognostic online tool (Predict:Breast Cancer) but adapted to a different scenario (kidney cancer) where the treatment options are simpler.

Below we briefly review the relevant theories and literature that have informed the design of the study.

### Uncertainty Communication and Trust

While the framework from van der Bles et al.^7^ on the communication of uncertainty identifies trust with the warmth-competence distinction,^12^ it is fully compatible with more explicit models of trust such as that of Mayer et al.,^10^ which distinguishes between two antecedents of trust (benevolence and ability) that map onto the warmth-competence model and includes another (integrity). These distinctions imply that uncertainty communication may affect different antecedents of trust differently: admitting uncertainty may increase perceived integrity while (at least for some people) decreasing perceived competence, as empirically demonstrated by Johnson and Slovic.^13^

Popular approaches to communicating uncertainty in prognostic tools include providing specific numbers with caveats about why they may not apply to particular cases, a range (e.g., “20%–30%”), or no uncertainty at all. However, research has frequently found null effects on perceptions of honesty, source credibility, source trust, or trustworthiness when using these approaches.^14–20^ In one study, a source’s discussion of uncertainty in the numbers made the source seem more honest to 66% of participants,^17^ but negative effects on the perceived credibility or trustworthiness of the source have been reported in some specific contexts, for example, when the amount of uncertainty is very large^18^ or when the quality of evidence is described as low.^20,21^

In sum, theory and experimental research do not necessarily anticipate consistent or strong effects of uncertainty communication on an audience’s trust in the source of the information, given the importance of contextual factors and the differential effects of uncertainty communication on perceived competence and perceived integrity. The picture becomes more complex when we consider that real-world online tools tend to be branded by an organization that vouches for them. Is a statement of uncertainty on a Web site (which may, in isolation, reduce perceptions of competence/ability) interpreted in the same way if the Web site is endorsed by institutions perceived as highly competent?

### Institutional Endorsements and Trust

Trust transference theory^8^ suggests that trust in a trusted entity is expected to “rub off” on an unknown entity to a degree dependent on the apparent degree of interaction between the organizations, their degree of perceived similarity, as well as the presence of visible indicators of an association between them (i.e., logos on Web sites), which is hypothesized to increase both the apparent degree of interaction and the degree of perceived similarity. Much empirical work supports the role of endorsements in trust transference: Jiang et al.^22^ found that logo familiarity and favorable disposition toward third-party certifications were key predictors of a generalized positive perception of third-party certification identifying logos, which in turn had a positive effect on trust.^22^ Subsequent work has found that positive effects on trust are obtained by some but not all logos,^23^ although the surprisingly powerful effects of even unfamiliar logos in some contexts have led other researchers to argue that “familiarity with particular details of a logo may be less important than a sense that the logo ‘seems’ familiar.”^24(p847)^ The familiarity and credibility of a third-party logo affects user trust in unfamiliar Web sites, even when logo exposure is brief.^25^

**Figure 1:**
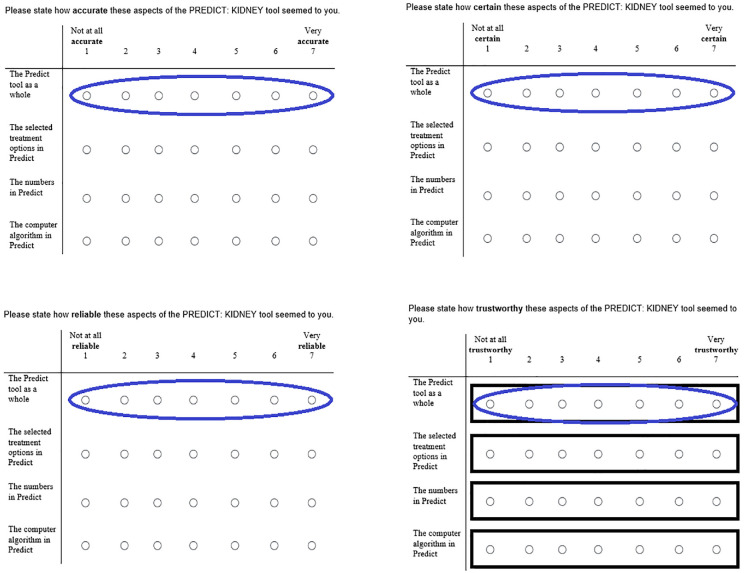
Illustration of the relationship between the items in the “perceived trustworthiness” measure, indicated by rectangles, and the measure of “trust in the tool as a whole”, indicated by ovals.

Beyond this, it is important to recognize that medical decisions take place in health care contexts and that decision-making preferences are also influenced by feelings of trust toward the medical profession generally, even more so than by trust in their own physician.^26^ This can be measured with the construct of generalized trust, described as “one’s general willingness to trust others,”^27(p49)^ in this case medical professionals, and “akin to a personality trait that a person would presumably carry from one situation to another,”^10(p715)^ as well as generalized institutional trust, a version of this construct that is specific to “people’s trust in the formal institutions and rules that regulate their lives,”^28(p10)^ (see also Rothstein^29^), in this case institutions connected with medical research and practice. Generalized trust and generalized institutional trust have been shown to be powerful predictors of the amount of trust that individuals end up placing in specific decision-relevant targets.^27,28^ In addition, we chose to investigate 2 further dimensions of trust directly connected to the online tool for decision making: a measure of how “trustworthy” participants perceived specific components (the treatment options, the numbers, the computer algorithm, and the tool as a whole) of the tool to be (together called “perceived trustworthiness”) as well as an attitudinal measure of the tool called “trust in the tool as a whole” (comprising measures of the “accuracy,” “certainty,” “reliability,” and “trustworthiness” of “the tool as a whole”; see Figure 1). This is based on previous work suggesting that these dimensions may be theoretically distinct in the context of trust toward technological artifacts. For example, Lee and See distinguished between cases of “high functional specificity,” in which “a person’s trust reflects capabilities of specific subfunctions and modes” of the technology, and “low functional specificity,” in which “the person’s trust reflects the capabilities of the entire system.”^30(p56)^

### Bringing Uncertainty Communication and Institutional Endorsements Together

There are reasons to expect that the effects of endorsements and uncertainty cues may interact. For example, Cazier^9(p49)^ argued that trust “produced with the interaction of a third party that vouches for the ability of the firm or individual providing the good or service,” which he refers to as “institution-based trust,” transfers differentially along Mayer et al.’s dimensions of ability, benevolence, and integrity. Depending on how an endorsing institution is perceived, its endorsement might increase perceptions of ability but not of benevolence, or the reverse. Van der Bles et al.^7^ speculated that in cases in which a communicator is perceived as high in competence (ability) but low in warmth (benevolence/integrity), communicating uncertainty might improve the latter without meaningfully reducing the former, providing the missing ingredient necessary to establish an adequate level of trust. If an institutional logo associated with a scientific or medical organization primarily increases impressions of competence, perhaps the combined effect of this logo and an uncertainty statement will be greater than the sum of their parts. However, the reverse could also be true: by the principle of least effort,^31^ individuals tend to process as little information as possible to reach a judgment and might merely ignore the less salient cue when both are present.

### Hypotheticality

Medical treatment decision scenarios necessarily involve hypothetical events. In construal level theory,^11^ the degree to which a situation feels hypothetical, fictional, or otherwise unreal is captured by the “hypotheticality” dimension of *psychological distance*. Psychological distance is a multidimensional construct that captures the degree to which a situation feels close or distant in space, time, social distance, and hypotheticality (“the distinction between real and imagined objects and between probable and improbable events”^11[p444]^). Hypotheticality encompasses considerations such as whether participants ever had to face real medical decisions in their lives similar to the ones presented in the hypothetical scenarios. The findings of various studies imply that trust tends to be higher when various dimensions or proxies of psychological distance are lower,^32–36^ but none have specifically looked at the effects of hypotheticality on trust. This is of particular importance given the number of studies that attempt to study trust in online surveys or in controlled laboratory environments rather than in more naturalistic settings. Although the use of clinical scenarios in online studies has benefits in terms of controllability of the environment, reproducibility, and the ease of collecting a large sample, hypotheticality is likely to be high. If feelings of hypotheticality have predictable and substantial effects on trust, it may be important for trust researchers to routinely measure it and adjust for its influence.

In this study, then, we conducted a pilot and 2 experiments (N_total_ = 4,724) using a fictional but realistic medical scenario in which participants were asked to imagine that they had recently received a diagnosis of localized renal cell carcinoma (kidney cancer) and were presented with a scenario describing 2 treatment options: monitoring only or partial nephrectomy (removal of part of the kidney). By using stimuli based on a real prognostic online tool (Predict:Breast Cancer, but modified for kidney cancer), we were able to provide a more realistic context for the decision than is typical for experiments based on hypothetical scenarios.

## Experiment 1

Before starting experiment 1, we conducted a pilot that made no specific hypotheses beyond an effect of uncertainty and institutional cues—as well as covariates such as numeracy and generalized institutional trust—on perceived trustworthiness, accuracy, reliability, and certainty of the prognostic online tool (Appendix 1). Based on its findings and on the theoretical considerations described above, we preregistered 6 specific hypotheses for experiments 1 and 2 and tested them in a UK and an international population, respectively.

### Hypotheses

We preregistered 6 hypotheses at https://osf.io/rc4me

H1: That the effect of institutional cue on perceived trustworthiness would be moderated by the presence/absence of uncertainty cues. This was based on the finding from the pilot and theoretical considerations.
H2: That the effect of institutional cue on trust in the tool as a whole would be moderated by the presence/absence of uncertainty cues. This was similarly based on the finding from the pilot and theoretical considerations.
H3: That the effect of institutional cue on 1) attitudes toward the Predict tool as a whole, 2) decision certainty, and 3) the weighting of the information in Predict in decision making would be mediated by psychological distance, institutional trust, and attitudes toward the individual components of the Predict tool and moderated by the presence of uncertainty cues and past experience with cancer. H3 was informed by the trends in the pilot study and theoretical considerations.
H4: That which institutional cue was shown would be predictive of the relative weight that participants say they placed on the Predict tool as a whole.
H5: That which institutional cue was shown would be predictive of the relative weight that participants say they placed on the institutions associated with the development of Predict.
H6: That participants who felt close to the situation (low psychological distance) would feel the least control over negative outcomes and therefore pay the least attention to institutional cues that provide trust-relevant information. We hypothesized that they would place less weight on institutions associated with the development of Predict and more weight on other factors.

### Methods

#### Participants

A power analysis indicated that 1,749 participants would be required to achieve 90% power to detect a small effect (*f* = 0.10) in perceived trustworthiness (H1) at α = 0.05. This effect size was chosen as it was just beneath the size of the effect detected in the pilot (*f* = 0.11). As we expected up to 5% of participants to fail the attention check, we intended to recruit 1,841 participants. Participants were recruited online using the platform Prolific.co and completed the survey hosted on Qualtrics. The only eligibility criteria were that participants must be 18 y or older (and, in experiment 1, a resident in the United Kingdom). Participants were paid £2.18 for their participation. A total of 1,845 individuals completed the experiment; 1,777 were analyzed after removing those who failed the attention check. Participant characteristics are reported in Supplemental Table S1.

#### Design

**Figure 2:**
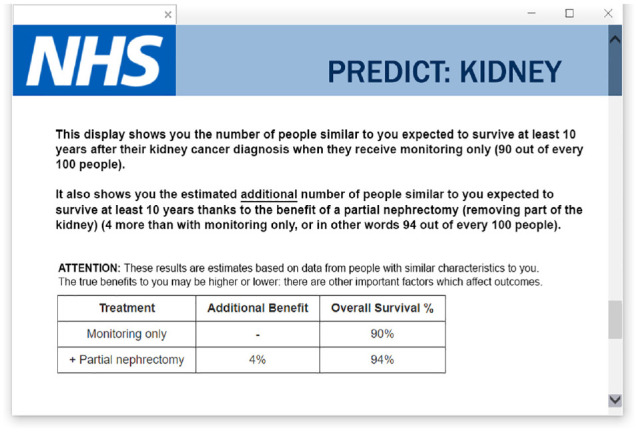
Example stimulus illustrating the National Health Service (NHS) logo and an uncertainty cue (experiment 1).

We used a 3 (*uncertainty cue*) × 4 (*institution cue*) between-participants factorial design. The 3 uncertainty cue conditions were no statement of uncertainty, a statement that “the true benefits to you may be higher or lower: there are other important factors which affect outcomes,” and a statement that “the tool’s estimates are less well tested for people with the characteristics you have selected” (based on statements currently used in the Predict:Breast Cancer and Predict:Prostate tools). The 4 institution cue conditions were no logo, the logo of Cambridge University, the logo of the United Kingdom’s National Health Service, or the logo of the pharmaceutical company GlaxoSmithKline (the University of Cambridge and NHS logo being those used on the real-life Predict tool). An example stimulus is illustrated in Figure 2. Apart from the difference in logos and uncertainty statements, the same stimulus was used for all participants, and it was made clear to participants that they were seeing fictionalized information (see Supplemental Table S2 for the actual survey instrument wording). The stimulus was shown to participants only once in each experiment, and no questions were asked while they could view it—only before and after.

### Measures

See Supplemental Table S2 for full details on measures.

#### Dependent measures

The 4 primary dependent variables were perceived accuracy, reliability, certainty, and trustworthiness of the prognostic online tool.

Each was measured via a 4-item measure that asked how accurate/reliable/certain/trustworthy participants found the tool as a whole, the treatment options displayed within it, the numbers in the tool, and the computer algorithm that produced the numbers. These responses were also used to build 4-item measures of participants’ trust in these 4 different aspects of the tool, yielding measures of participant trust in the tool as a whole, the treatment options, the numbers, and the computer algorithm. The numeric responses to each of the 4 items in a measure (Figure 1) were averaged to yield a participant’s final score for that measure.

We collected participants’ hypothetical treatment decisions (monitoring only or partial nephrectomy) before and after viewing the tool, allowing us to look at the proportion of participants who switched their decision.

We also measured decision confidence as well as how much weight participants reported placing on the information provided by the tool when making their decision and the proportion of this weight that participants reported placing on different information subtypes. Decision confidence was also measured before and after viewing the tool, providing a measure of change in decision confidence.

Finally, we included exploratory measures of worry about treatment options, gist recall, verbatim recall, and information sufficiency (whether participants felt they had all the information they needed to make a decision).

#### Covariates

Generalized institutional trust was measured as the mean of answers to the following Likert items: 1) I trust health care professionals in the UK, 2) I trust the NHS, 3) I trust universities in the UK, 4) I trust the University of Cambridge, 5) I trust scientists, 6) I trust scientific knowledge, 7) I trust doctors, 8) I trust my own doctor, 9) I trust the pharmaceutical industry, and 10) I trust GlaxoSmithKline. For each, participants indicated their agreement on a 7-point scale ranging from *strongly disagree* (1) to *strongly agree* (7). The measure was composed of the same 10 items irrespective of experimental condition. Familiarity with each institution was measured with 3 Likert items asking, “How familiar are you with the University of Cambridge?”, “How familiar are you with the NHS?”, and “How familiar are you with GlaxoSmithKline?”, ranging from 1 (*not at all*) to 7 (*very much*). For participants not randomized to the “no institution cue” control condition, we used their answers to the relevant institutions on the generalized institutional trust scale to construct an index of trust in the cued institution and to the relevant familiarity question to construct an index of familiarity with the cued institution.

We also included covariates to measure psychological distance: a scale of belief modified from Fishbein and Raven,^37^ intended to capture the “hypotheticality” dimension in construal level theory,^11^ and a 7-point, 1-item scale intended to capture social distance: “Have you ever had to weigh up options in real life in a way that is at all similar to the situation presented in this study?”

Numeracy was assessed with the Berlin adaptive numeracy test^38^ plus 3 additional numeracy questions based on Schwartz et al,^39^ as recommended by Cokely et al.^40^ We used the Decision Making Preference Questionnaire^41^ and recorded participants’ self-reported level of digital literacy along with demographic variables.

Finally, whereas the pilot used the question, “Were there any institutional logos associated with the tool?” as an attention check (with participants answering incorrectly being excluded), we instead included a question in which the correct answer was not correlated with the condition the participant was assigned to (“How worried would we be if you didn’t pay attention? To check that you are paying attention, please select ‘somewhat agree’ below”). Participants who failed the attention check question were excluded (as specified in the preregistration). Other ways in which the pilot differed from the measures described in this section are described in Appendix 1.

### Procedure

Participants were presented with the decision scenario; the full text is provided in rows 1 to 4 of Supplemental Table S2. We then collected initial treatment decision (monitoring only or partial nephrectomy) with the question, “If you had to make a choice with only the information you have learned up to now, what would you choose?” and decision confidence as described. This was followed by a continuation of the scenario in which the doctor proceeded “to show you an online tool that some patients find helpful when deciding together with their clinicians which treatments to have” and explained the purpose of the tool. Participants were then shown the stimulus corresponding to the particular condition to which they had been randomized (e.g., Figure 2). This was followed by 4 recall questions and the scales for perceived accuracy, certainty, reliability, and trustworthiness. Afterward, participants were again asked, “If you had to make a choice with only the information you have learned up to now, what would you choose?” Finally, the remaining measures were collected (Supplemental Table S2). The funding body had no input into the design of the experiments.

### Statistical Analysis

For each of H1 and H2, we preregistered 4 × 3 factorial analyses of variance (ANOVA). In each case, the statistical model tested the main effects of institutional cue and uncertainty cue, as well as their interaction, on the dependent variable (H1: perceived trustworthiness; H2: trust in the tool as a whole). For H3, we preregistered the intent to conduct “exploratory mediation analysis with on attitudes towards the Predict tool as a whole, decision certainty, and the weighting of the information in Predict as DVs” and specified possible mediators and moderators. For each of these dependent variables, we opted to conduct 2 separate single-mediator models with psychological distance or generalized institutional trust as the mediator, respectively, and to run additional analyses including moderators only if there was a significant effect to be explained. For H4, H5, and H6, we preregistered our intent to run regression analyses to see whether psychological distance, and the particular institutional cues participants were shown, were predictive of the weight participants reported placing on the information provided by the Predict tool (H4) and the relative weight participants said they reported placing on each aspect of the Predict tool. One of these was the institutions associated with the development of Predict (referenced in H5, H6). Because specific covariates were not preregistered, we opted to additionally run multiple regression models with various combinations of covariates. The models used to investigate H4 used the reported weight placed on the information provided by the Predict tool as the dependent variable. The models used to investigate H5 and H6 each used the weight that participants indicated that they placed on the institutions associated with the development of Predict as the dependent variable. Sum coding was used for institutional cues (4 levels) and uncertainty cues (3 levels), which were treated as unordered factors. The models used to investigate H4 and H5 contained the following independent variables, respectively, as fixed effects:

1. the 2 manipulated variables (institutional cue and uncertainty cue) only;
2. the 2 manipulated variables + generalized institutional trust;
3. the 2 manipulated variables + hypotheticality; and
4. the 2 manipulated variables + their interaction (equivalent to factorial ANOVA, as we are using sum coding and ordinary least squares).

We refer to the first of these as the “base model”. For H6, because the hypothesis pertained to hypotheticality, only the model containing hypotheticality was used.

For H4 and H5, results from an ANOVA mathematically equivalent to the model (4) above are reported in the main text. This approach allowed us to collapse the multiple coefficients associated with the institutional cue variable—which has 4 levels—into a single significance test and effect size, streamlining the presentation of findings. (Conditional on the experiment and the hypothesis, the results of all 4 models were always consistent with the ANOVA—i.e., institutional cue in the ANOVA was significant when and only when some institutional cue was significant across all 4 regression models and was insignificant when and only when no institutional cue was significant across all 4 regression models.) All other analyses reported were exploratory. Type III sum of squares was used for ANOVAs that included interactions, while type II was used for those without interactions.

### Results

The 68 participants failing the attention check were excluded, leaving 1,777 participants for analysis (Table 1). ANOVA results are reported in Table 2. H1 and H2 were not supported, as there was no interaction for trust in the tool as a whole nor perceived trustworthiness, although there was a main effect of institutional cue in both cases. These main effects were followed up with Tukey’s post hoc tests. For both variables, only the difference between GlaxoSmithKline and Cambridge was significant. Single-mediator models did not find direct or indirect effects of the presence (v. absence) of an institutional cue on trust in the tool as a whole, decision certainty, or weighting of the information in Predict, failing to support H3.

**Table 1:** Number of Participants Analyzed in Each Condition in the Pilot and Experiments

**Pilot (Appendix 1)**	**University of Cambridge**		**National Health Service**	**No Institution Cue**	**Total**
“PREDICT is based on data from a selection of hospitals”	103		115	80	298 (33.9%)
“PREDICT only displays an estimated average benefit”	103		108	75	286 (32.5%)
No uncertainty cue	106		110	79	295 (33.6%)
Total	312 (35.5%)		333 (37.9%)	234 (26.6%)	
	**University of Cambridge**	**National Health Service**	**GlaxoSmithKline**	**No Institution Cue**	**Total**
Experiment 1					
No uncertainty cue	148	148	148	149	593 (33.4%)
“There are other important factors which affect outcomes”	150	147	145	147	589 (33.1%)
“The tool’s estimates are less well tested . . .”	149	147	147	152	595 (33.5%)
Total	447 (25.2%)	442 (24.9%)	440 (24.8%)	448 (25.2%)	
Experiment 2					
No uncertainty cue	171	178	179	177	705 (34.1%)
“There are other important factors which affect outcomes”	172	168	169	170	679 (32.8%)
“The tool’s estimates are less well tested . . .”	170	170	174	170	684 (33.1%)
Total	513 (24.8%)	516 (25.0%)	522 (25.2%)	517 (25.0%)	

**Table 2:** Preregistered and Exploratory Analyses of Variance (ANOVAs), Experiment 1

	Uncertainty Cues			Institutional Cues			Uncertainty Cue × Institutional Cue Interaction		
	*F*	*P*	η^2^_G_	*F*	*P*	η^2^_G_	*F*	*P*	η^2^_G_
Preregistered ANOVAs									
Trustworthiness	0.18	0.83	0.000	3.32	0.019*	0.006	0.90	0.50	0.003
Trust in tool as a whole	0.91	0.40	0.001	2.86	0.036*	0.005	1.15	0.33	0.004
Exploratory ANOVAs									
Certainty	0.80	0.45	0.001	1.68	0.170	0.003	1.09	0.36	0.004
Accuracy	0.13	0.88	0.000	1.80	0.145	0.003	1.73	0.110	0.006
Reliability	0.43	0.65	0.000	1.36	0.25	0.002	1.78	0.099	0.006
Trust in treatments	0.36	0.70	0.000	2.18	0.088	0.004	0.98	0.44	0.003
Trust in numbers	0.36	0.70	0.000	1.06	0.37	0.002	1.67	0.126	0.006
Trust in algorithm	0.25	0.78	0.000	1.99	0.113	0.003	1.53	0.163	0.005
Weight placed on tool	1.58	0.21	0.002	2.44	0.063	0.004	0.53	0.79	0.002
% wt on numbers	1.56	0.21	0.002	7.42	<0.001***	0.012	0.46	0.84	0.002
% wt on institutions	0.21	0.81	0.000	64.18	<0.001***	0.098	0.55	0.77	0.002
% wt on algorithm	0.23	0.79	0.000	4.46	0.004**	0.008	1.26	0.27	0.004
% wt on data	3.13	0.044*	0.004	11.57	<0.001***	0.019	1.61	0.139	0.005
% wt on other	0.11	0.90	0.000	0.06	0.98	0.000	0.26	0.95	0.001
Worry	0.17	0.85	0.000	0.82	0.48	0.001	0.42	0.87	0.001
Information sufficiency	4.00	0.018*	0.005	4.14	0.006**	0.007	0.83	0.55	0.003
Decision confidence	1.08	0.34	0.001	1.02	0.38	0.002	0.80	0.57	0.003
Change in decision confidence	0.94	0.39	0.001	1.60	0.188	0.003	0.61	0.72	0.002

* *P* < 0.05; **P < 0.01; ***P < 0.001. *P*-values less than 0.05 are indicated in bold.

**Figure 3:**
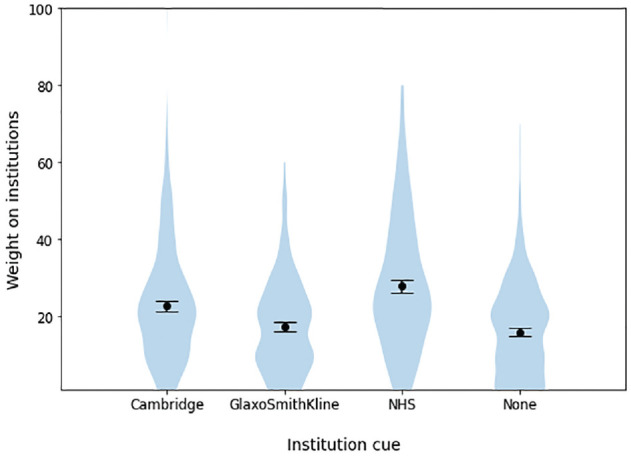
Proportion of weight that participants reported placing on “the institutions behind the development of Predict” across different institution cue conditions, experiment 1.

**Figure 4:**
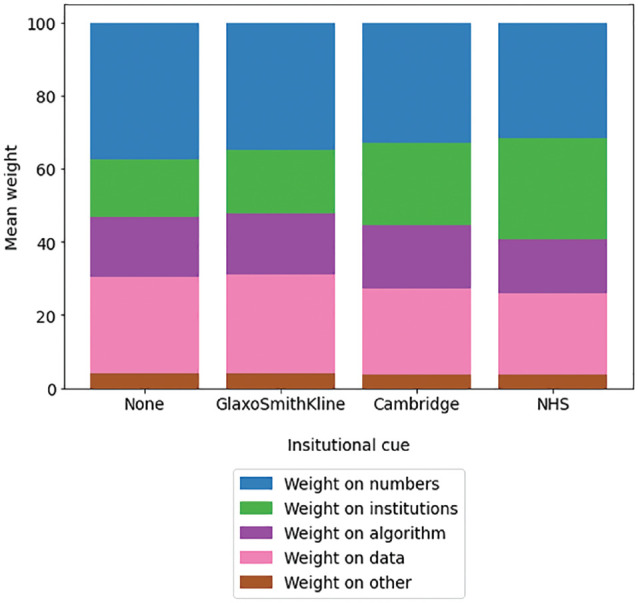
Mean proportion of weight that participants reported placing on “the institutions behind the development of Predict” across different institution cue conditions, experiment 1.

**Figure 5:**
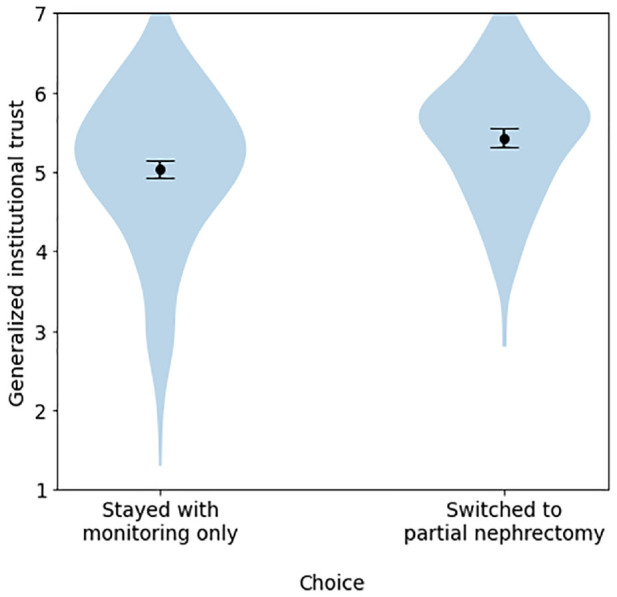
Differences in generalized institutional trust between participants who stayed with monitoring only versus those who switched to partial nephrectomy, among participants who initially chose monitoring only, experiment 1.

Institutional cues did not meaningfully predict the overall amount of weight that participants reported placing on the Predict tool (failing to support H4), with a negligible effect that failed to reach significance (η^2^_G_ = 0.004, *P* = 0.063). However, as in the pilot, they predicted the proportion of this weight that participants reported placing on institutions, supporting H5 (η^2^_G_ = 0.098; Table 2, Figures 3 and 4). The presence or absence of a specific institutional logo, as well as the particular uncertainty message shown, had minimal influence on the outcomes measured. In contrast, generalized institutional trust and trust in the cued institution were stronger predictors of most dependent variables, including trustworthiness and trust in the tool as a whole (Tables 3 and 4). This same pattern of effects was observed in the pilot study (see Appendix 1). By contrast, hypotheticality and the proportion of weight participants reported placing on institutions were highly associated (B = 1.18, *P* < 0.001), supporting H6. Directionally, the model using hypotheticality as a covariate explained more variance than the model using generalized institutional trust for all variables other than *worry* and *proportion of weight on algorithm*, although these differences were not always significant. The additional single-item measure of psychological distance, aimed at determining whether participants ever had to make a similar decision in real life, exhibited a severe ceiling effect; hence, future references to “psychological distance” refer solely to our measure of hypotheticality. Logistic regressions again found that generalized institutional trust and trust in the cued institution predicted the propensity to switch to active treatment (Tables 5 and 6, Figure 5).

**Table 3:** Coefficients and *P* Values for Covariates in Linear Regression Models across All Participants (*N* = 1,777), Experiment 1^a^

Model	Base Model (All Participants)	Base Model + Generalized Institutional Trust (All Participants)			Base Model + Hypotheticality (All Participants)		
	Model	Generalized Institutional Trust		Model	Hypotheticality		Model
	Adjusted *r* ^2^	B	*P*	Adjusted *r* ^2^	B	*P*	Adjusted *r* ^2^
Primary measures							
Trustworthiness	0.003	**0.39**	<0.001***	0.091	**−0.46**	<0.001***	0.160
Certainty	0.001	**0.27**	<0.001***	0.050	**−0.43**	<0.001***	0.171
Accuracy	0.000	**0.27**	<0.001***	0.061	**−0.42**	<0.001***	0.188
Reliability	−0.000	**0.34**	<0.001***	0.075	**−0.46**	<0.001***	0.172
Exploratory measures							
Trust in tool as a whole	0.003	**0.33**	<0.001***	0.081	**−0.43**	<0.001***	0.182
Trust in treatments	0.001	**0.30**	<0.001***	0.068	**−0.44**	<0.001***	0.185
Trust in numbers	−0.001	**0.32**	<0.001***	0.060	**−0.45**	<0.001***	0.154
Trust in algorithm	0.001	**0.33**	<0.001***	0.067	**−0.45**	<0.001***	0.166
Weight placed on tool	0.003	**2.45**	<0.001***	0.011	**−3.28**	<0.001***	0.021
% wt on numbers	0.011	−0.25	0.65	0.011	**−1.73**	<0.001***	0.018
% wt on institutions	0.096	**1.28**	<0.001***	0.101	**1.18**	<0.001***	0.102
% wt on algorithm	0.005	−0.17	0.53	0.005	0.04	0.88	0.004
% wt on data	0.020	0.07	0.87	0.019	−0.54	0.119	0.021
% wt on other	−0.003	**−0.93**	0.002**	0.002	**1.05**	<0.001***	0.005
Worry	−0.001	**−0.22**	<0.001***	0.034	**0.12**	<0.001***	0.012
Information sufficiency	0.009	**0.37**	<0.001***	0.044	**−0.43**	<0.001***	0.071
Decision confidence	0.000	**0.19**	<0.001***	0.032	**−0.19**	<0.001***	0.045
Change in decision confidence	0.001	0.04	0.073	0.002	**−0.06**	0.002**	0.006

a Each model included uncertainty cue, institutional cue, and a single covariate. *P < 0.05; **P < 0.01; ***P < 0.001. Coefficients and *P*-values where *P*-values are less than 0.05 are indicated in bold.

**Table 4:** Coefficients and *P* Values for Covariates in Linear Regression Models across Participants Shown Institutional Cues (*n* = 1,329), Experiment 1^a^

Model	Base Model (Participants Shown Institutional Cues Only)	Base Model + Trust in Cued Institution (Participants Shown Institutional Cues Only)			Base Model + Familiarity with Cued Institution (Participants Shown Institutional Cues Only)			Base Model + Generalized Institutional Trust (Participants Shown Institutional Cues Only)		
	Model	Trust in Cued Institution		Model	Familiarity with Cued Institution		Model	Generalized Institutional Trust		Model
	Adjusted *r* ^2^	B	*P*	Adjusted *r* ^2^	B	*P*	Adjusted *r* ^2^	B	*P*	Adjusted *r* ^2^
Primary measures										
Trustworthiness	0.005	**0.25**	<0.001***	0.080	**0.04**	0.039*	0.007	**0.39**	<0.001***	0.095
Certainty	0.001	**0.16**	<0.001***	0.036	0.02	0.24	0.001	**0.27**	<0.001***	0.050
Accuracy	0.002	**0.16**	<0.001***	0.043	**0.04**	0.035*	0.004	**0.27**	<0.001***	0.063
Reliability	0.002	**0.22**	<0.001***	0.062	0.03	0.127	0.003	**0.34**	<0.001***	0.077
Exploratory measures										
Trust in tool as a whole	0.006	**0.20**	<0.001***	0.065	**0.04**	0.013*	0.009	**0.32**	<0.001***	0.083
Trust in treatments	0.002	**0.18**	<0.001***	0.051	0.03	0.096	0.004	**0.31**	<0.001***	0.073
Trust in numbers	−0.000	**0.19**	<0.001***	0.044	0.03	0.111	0.001	**0.31**	<0.001***	0.058
Trust in algorithm	0.002	**0.21**	<0.001***	0.060	0.02	0.24	0.002	**0.33**	<0.001***	0.072
Weight placed on tool	0.003	**2.01**	<0.001***	0.014	0.40	0.33	0.003	**2.26**	0.002**	0.009
% wt on numbers	0.004	−0.57	0.173	0.004	−0.38	0.26	0.004	0.14	0.81	0.003
% wt on institutions	0.074	**2.10**	<0.001***	0.102	**0.87**	<0.001***	0.081	**1.62**	<0.001***	0.081
% wt on algorithm	0.009	−0.04	0.86	0.008	−0.07	0.70	0.008	−0.23	0.45	0.008
% wt on data	0.021	**−0.71**	0.024*	0.024	−0.47	0.059	0.023	−0.15	0.74	0.021
% wt on other	−0.002	**−0.78**	0.001**	0.005	0.05	0.80	−0.003	**−1.38**	<0.001***	0.009
Worry	−0.002	**−0.15**	<0.001***	0.029	−0.02	0.170	−0.002	**−0.22**	<0.001***	0.034
Information sufficiency	0.008	**0.22**	<0.001***	0.035	**0.07**	0.015*	0.012	**0.35**	<0.001***	0.040
Decision confidence	−0.002	**0.10**	<0.001***	0.019	0.03	0.054	0.000	**0.16**	<0.001***	0.022
Change in decision confidence	0.001	**0.04**	0.017*	0.004	0.01	0.57	0.000	0.03	0.171	0.001

a Each model included uncertainty cue, institutional cue, and a single covariate. *P < 0.05; **P < 0.01; ***P < 0.001. Coefficients and *P*-values where *P*-values are less than 0.05 are indicated in bold.

**Table 5:** Coefficients and *P* Values for Covariates in Logistic Regression Models Including Uncertainty Cue, Institutional Cue, and a Single Covariate for Exploratory Binary Measures, Experiment 1^a^

	Model						
	Institutional, Uncertainty Cues Only (All Participants)	Institutional, Uncertainty Cues + Generalized Institutional Trust (All Participants)			Institutional, Uncertainty Cues + hypotheticality (All Participants)		
		Generalized Institutional Trust			Hypotheticality		
Dependent variable	Pseudo *r* ^2^	Pseudo *r* ^2^	B	*P*	Pseudo *r* ^2^	B	*P*
Switched their choice after viewing tool (switched = 1); *n* = 1,777	0.005	0.005	0.07	0.32	0.005	−0.02	0.79
Switched to active treatment after viewing tool (switched = 1) (only those who initially chose passive treatment: *n* = 516)	0.009	0.040	0.49	<0.001***	0.012	−0.13	0.155
Switched to passive treatment after viewing tool (switched = 1) (only those who initially chose active treatment: *n* = 1,261)	0.005	0.006	−0.11	0.23	0.005	0.05	0.56

a McFadden’s^42^ pseudo *R* ^2^s are simply 1 minus the ratio of the model’s log-likelihood to that of a model with intercept only and reflect the variance accounted for by the logistic regression model. *P < 0.05; **P < 0.01; ***P < 0.001. Coefficients and *P*-values where *P*-values are less than 0.05 are indicated in bold.

**Table 6:** Coefficients and *P* Values for Covariates in Logistic Regression Models Including Uncertainty Cue, Institutional Cue, and a Single Covariate for Exploratory Binary Measures, Experiment 1, Restricted to Participants Who Were Shown an Institutional Cue^a^

	Model									
	Institutional, Uncertainty Cues Only	Institutional, Uncertainty Cues + Trust in Cued Institution			Institutional, Uncertainty Cues + Familiarity with Cued Institution			Institutional, Uncertainty Cues + Generalized Institutional trust		
		Trust in Cued Institution			Familiarity with Cued Institution			Generalized Institutional Trust		
Dependent Variable	Pseudo *r* ^2^	Pseudo *r* ^2^	B	*P*	Pseudo *r* ^2^	B	*P*	Pseudo *r* ^2^	B	*P*
Switched their choice after viewing tool (switched = 1) (only those who were shown an institutional cue: *n* = 1,329)	0.005	0.008	0.10	0.074	0.006	0.02	0.59	0.006	0.10	0.22
Switched to active treatment after viewing tool (switched = 1) (only those who initially chose passive treatment and were shown an institutional cue: *n* = 377)	0.012	0.030	**0.26**	0.004**	0.014	−0.08	0.28	0.055	**0.58**	<0.001***
Switched to passive treatment after viewing tool (switched = 1) (only those who initially chose active treatment and were shown an institutional cue: *n* = 952)	0.005	0.006	0.05	0.48	0.009	0.10	0.075	0.006	−0.09	0.41

a McFadden’s^42^ pseudo *R* ^2^s are simply 1 minus the ratio of the model’s log-likelihood to that of a model with intercept only and reflect the variance accounted for by the logistic regression model. *P < 0.05; **P < 0.01; ***P < 0.001. Coefficients and *P*-values where *P*-values are less than 0.05 are indicated in bold.

Institution and uncertainty cues were not predictive of any binary measures. Details and additional exploratory analyses are reported in the Supplementary Materials.

### Discussion

Experiment 1 provided a clear replication of some of the findings of the pilot and a failure to replicate others. An interaction between uncertainty and institution cues observed in the pilot failed to replicate with the modified attention check. Institution cues also failed to explain substantial variance in the amount of weight that participants said that they placed on the tool when considering a hypothetical decision about treatment. However, these cues were not ignored: in both the pilot and experiment 1, generalized institutional trust and trust in the cued institution had a substantial effect on the proportion of weight that participants said they placed on the institutions endorsing the tool.

Do generalized institutional trust and trust in the cued institution both matter? In the pilot, unique variance had been attributable to generalized institutional trust and trust in the cued institution for each of the primary measures (certainty, reliability, accuracy, trustworthiness) and each of the “trust” dimensions (trust in the tool as a whole, trust in the treatment options, trust in the numbers, and trust in the algorithm; Supplemental Table S5). The same general pattern was observed in experiment 1, although less consistently: trust in the cued institution was not a significant predictor for certainty, accuracy, or trust in treatment options (Supplemental Table S6). Overall, the greater variance accounted for by generalized institutional trust on these variables in experiment 1 and the pilot, irrespective of whether these variables were entered into regressions together or separately (Appendix Table A2; Table 4; Supplemental Tables S5 and S6), suggests it is the more important predictor.

Both generalized institutional trust and trust in the cued institution were predictors of the proportion of weight that participants reported placing on institutions. However, the models with trust in institution explained more variance for this dependent variable than the models with generalized institutional trust (Appendix Table A2; Table 4), and when including both predictors in the same model, trust in the cued institution was the only significant predictor (Supplemental Tables S5 and S6). Although the coefficients in this model may be unstable due to modest collinearity between these predictors (*r* = 0.73 and *r* = 0.62 in the pilot and experiment 1, respectively), the consistency in both experiments nevertheless suggests that trust in the cued institution may be driving this effect.

For participants who had initially chosen passive treatment, generalized institutional trust and trust in the cued institution also predicted propensity to switch treatment after viewing the tool in both (Appendix Tables A2 and A3; Tables 5 and 6). Models with generalized institutional trust explained a higher proportion of the variance (Appendix Table A3; Table 6). Furthermore, in a model that included both generalized institutional trust and trust in cued institution as predictors, only the former was significant; that said, these variables were correlated, and models including both variables did not find this effect in the other (Supplemental Tables S8–S10).

Hypotheticality predicted weight on institutions as well as trustworthiness, certainty, accuracy, reliability, and measures of trust (in numbers, treatments, the algorithm, and the tool as a whole). Individuals who were more skeptical of the scenario (high hypotheticality) were less trusting of all aspects of the tool. With respect to these variables, hypotheticality explained more variance than any other single predictor.

## Experiment 2

In experiment 2, we tested the same hypotheses as in experiment 1 on an international sample, recruited through the same platform. We reasoned that the combination of these experiments would help determine whether the relationships we saw between trust and familiarity in the institutions and our dependent variables were robust. The real-life Predict tools are used internationally but carry the logos of the UK-based institutions that are associated with them.^43^ Methods were as in experiment 1, except that we aimed to recruit 2,187 participants to ensure adequate power even if as many as 20% of participants were to fail the attention check. Full participant characteristics are given in Supplemental Table S1. Experiment 2 was preregistered at https://osf.io/fq5e7.

### Results

A total of 2,185 individuals completed the experiment, with *n* = 2,068 after participants failing the attention check were removed. Supplemental Table S11 reports the descriptives, Supplemental Table S12 reports the ANOVA results, and a comparison of the findings of each experiment with respect to our preregistered hypotheses appears in Table 7. In Table 8 we also consider how the hypotheses and findings correspond to theoretical propositions based on the literature discussed in the introduction.

**Table 7:** Comparative Analysis of Experimental Findings for the Preregistered Hypotheses

Hypothesis	Supported in Experiment 1	Supported in Experiment 2
H1: The effect of institutional cue on perceived trustworthiness will be moderated by the presence/absence of uncertainty cues	No	No
H2: We predicted that the effect of institutional cue on trust in the tool as a whole will be moderated by the presence/absence of uncertainty cues	No	No
H3: The effect of institutional cue on attitudes toward the Predict tool as a whole, decision certainty, and the weighting of the information in Predict in decision making will be mediated by factors such as psychological distance, institutional trust, and attitudes toward the individual components of the Predict tool and moderated by factors such as the presence of uncertainty cues and past experience with cancer	No	No
H4: Which institutional cue was shown will be predictive of the relative weight that participants say they placed on the Predict tool as a whole	No	No
H5: Which institutional cue was shown will be predictive of the relative weight that participants say they placed on the institutions associated with the development of Predict	Yes	Yes
H6: Participants with low psychological distance to the situation place less weight on institutions associated with the development of Predict	Yes	No

**Table 8:** Comparative Analysis of Experimental Findings More Generally

Proposition from Theory	Supported in Pilot	Supported in Experiment 1	Supported in Experiment 2	Relevant Tables
Proposition 1. The level of trust an individual has in an endorsing institution will be associated with how trustworthy they perceive the endorsed tool to be	Yes	Yes	Yes	Table A2, Table 4, Table S14
Proposition 2. Uncertainty cues may affect perceived trustworthiness	Yes (interaction; no main effect)	No	Yes (main effect only)	Table A1, Table 2, Table S12
Proposition 3. (a) Participants who are shown a prognostic tool labeled with an institutional logo place more weight on that tool when making decisions that the tool is intended to inform (b); (b) participants shown a tool with an institutional logo are expected to place more weight on the institutions endorsing the tool when making their decisions, compared with being shown a tool without any institutional logo	(a) Yes; (b) Yes	(a): No (but see Prop 4 below); (b): Yes	(a): No (but see Prop 4 below); (b): Yes	Tables A1, Table 2, Table S12
Proposition 4. The amount of weight that participants place on the tool in their decision making will be associated with (a) trust in an institution whose logo appears on a prognostic online tool and (b) generalized institutional trust	(a) No; (b) Yes	(a) Yes; (b) Yes	(a) Yes; (b) Yes	Tables A2, Table 3, Table 4, Table S13, Table S14 Further substantiation that (hypothetical) decision making is associated with trust in the institution whose logo appears and generalized institutional trust: Table A3, A4, Table 5, Table 6, Table S15, Table S16
Proposition 5: Hypotheticality of a medical decision scenario is inversely associated with trust	Not tested	Yes	Yes	Table 3, Table S13
Proposition 6: Participants for whom psychological distance is lower place less weight on the institutions endorsing the prognostic tool	Not tested	Yes	No	Table 3, Table S13

As in experiment 1, there was no interaction in the 2 preregistered ANOVAs. However, there was a very weak (η^2^_G_ = 0.003) main effect of uncertainty cues on trust in the tool as a whole, with Tukey’s post hoc tests suggesting marginally lower trust when the “The tool’s estimates are less well tested. . . ” cue was used (mean 4.95, 95% confidence interval [CI] 4.87–5.02) versus no uncertainty cue (mean 5.08, 95% CI 5.00–5.15). Main effects of uncertainty cue were also found for accuracy, reliability, and trust in the numbers, although these were just as weak (each η^2^_G_ ≈ 0.003). Tukey’s post hoc tests consistently found no significant differences (reliability, trust in the numbers) or a significant difference but with overlapping confidence intervals and a very small mean difference (accuracy, with “The tool’s estimates are less well tested . . .,” mean 4.94, 95% CI 4.87–5.01; v. “There are other factors which affect outcomes,” mean 5.06, 95% CI 4.99–5.13). There was a main effect of institutional cue on the proportion of weight that participants said they placed on institutions (η^2^_G_ = 0.029), with the highest proportion of weight placed there when the cue was the University of Cambridge (mean 22.6%, 95% CI 21.4%–23.8%) or the NHS (mean 21.8%, 95% CI 20.6%–22.9%), followed by GlaxoSmithKline (mean 19.3%, 95% CI 18.1%–20.5%) and then no logo at all (mean 16.5%, 95% CI 15.3%–17.7%). Single-mediator models using hypotheticality and institutional trust as mediators (respectively) did not find direct or indirect effects of the presence (v. absence) of an institutional cue on trust in the tool as a whole, decision certainty, or weighting of the information in Predict.

Additional exploratory analyses are reported in the Supplementary Materials. We again found that generalized institutional trust, hypotheticality, and trust in the cued institution accounted for much more variance than institutional or uncertainty cues (Tables S13–S14); in addition, familiarity with the cued institution was more predictive of most dependent variables in this study than in experiment 1, although it still accounted for far less variance on uncertainty measures or trust measures than did trust in the cued institution. We again observed that generalized institutional trust and trust in the cued institution predicted the propensity to switch to active treatment (Supplemental Tables S15–S16).

### Discussion

As in experiment 1, the results did not support H1 to H4 but showed clear support for H5: institutional cues predicted the proportion of weight participants said they placed on the institutions associated with the development of the tool in the pilot and both experiments. H6, which predicted that participants with more psychological distance from the situation described would place more weight on institutions associated with the development of the tool—was borne out in experiment 1 (*P* < 0.001) but was not significant in experiment 2 (*P* = 0.071). In any event, hypotheticality explained an extremely small amount of variance (<1%) in both cases.

**Figure 6:**
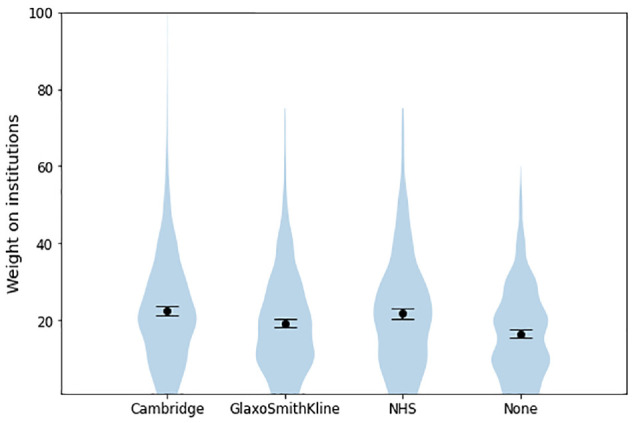
Proportion of weight that participants reported placing on “the institutions behind the development of Predict” across different institution cue conditions, experiment 2.

**Figure 7:**
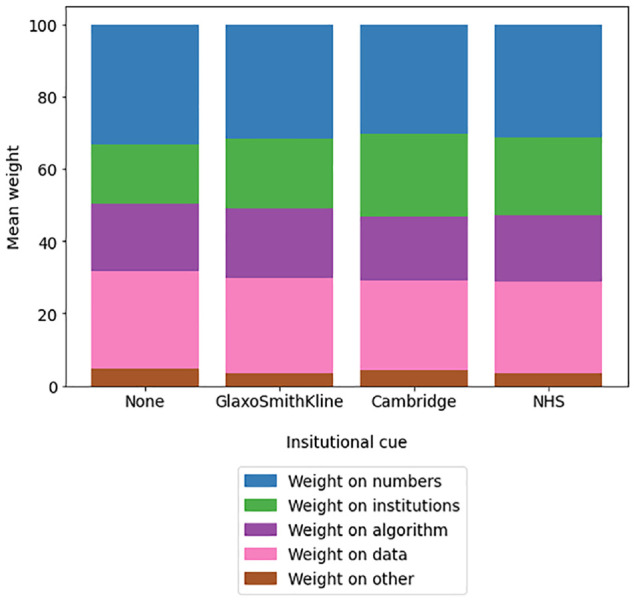
Mean proportion of weight that participants reported placing on “the institutions behind the development of Predict” across different institution cue conditions, experiment 2.

**Figure 8:**
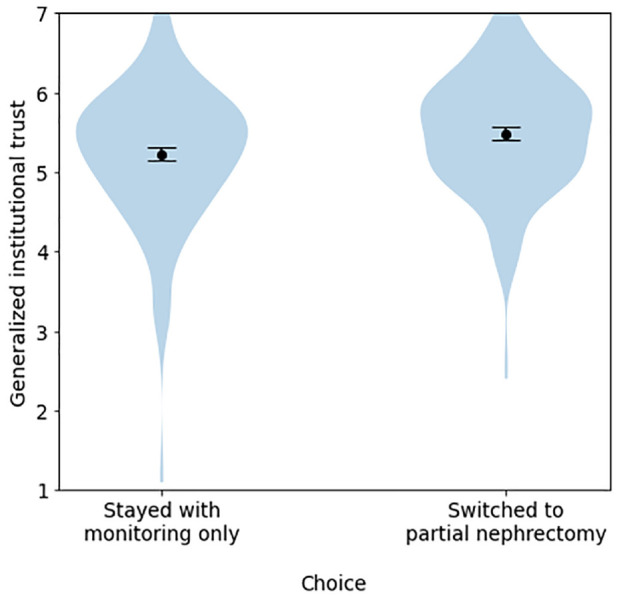
Differences in generalized institutional trust between participants who stayed with monitoring only versus those who switched to partial nephrectomy, among participants who initially chose monitoring only, experiment 2.

Once again, the strongest and most consistent findings were the effects of generalized institutional trust on trustworthiness, certainty, accuracy, reliability, and on trust in numbers, treatments, the algorithm and in the tool as a whole (Supplemental Table S13). As in experiment 1, generalized institutional trust tended to explain more variance in these models than did trust in the cued institution (Supplemental Table S14) and did so more consistently when both variables were entered into the same model (Supplemental Table S7), suggesting that the former was the driving factor. On the other hand, trust in the cued institution seemed to be a stronger predictor of the proportion of weight that participants said they placed on the institutions involved with the tool (Supplemental Tables S6–S7; Figures 6 and 7), in line with the pilot and experiment 1. As in those cases, generalized institutional trust and trust in the cued institution also each predicted the propensity to switch to partial nephrectomy for participants whose original treatment choice was monitoring only (Supplemental Tables S15–S16; Figure 8).

Hypotheticality was again a stronger predictor of certainty, reliability, accuracy, trustworthiness, and trust in the tool as a whole, the numbers, the algorithm, and the treatment options, relative to uncertainty and institution cues. It was also once again associated with lower ratings of information sufficiency and decision confidence and higher levels of worry (Supplemental Table S13).

## General Discussion

Across a pilot and 2 well-powered experiments that used an algorithmic tool and decision scenario based on an actual prognostic online tool (Predict:Breast Cancer, adapted to Predict:Kidney Cancer for the experiments) and employed a novel theoretical approach, we addressed issues faced by both researchers and designers of online, algorithmically driven tools: 1) the relevance of institutional endorsements and cues of organizations standing behind the online tool, 2) the relevance of uncertainty communication and cues about the data quality and reliability of algorithms, 3) the role of psychological distance (hypotheticality of scenario) for online trust, and 4) the relevance of all 3 previous aspects for the trust and subsequent decision making of general public users.

Our 3 measures of epistemic trust (perceived trustworthiness, trust in the tool as a whole, and the amount of weight that participants said they placed on the tool when making their decisions) were not robustly or consistently affected by the presence of uncertainty statements or by the presence of institutional logos per se. However, perhaps not surprisingly, institutional logos were powerful predictors of how much weight participants said they placed on the institutions behind the development of the tool when making their treatment decisions in the UK samples. A weaker association was seen in the experiment with the international sample, composed of participants who were less familiar with the UK institutions used in the stimuli.

Although participants’ level of trust in the particular organization whose logo was shown predicted the amount of weight placed on institutions better than a more general measure of trust in institutions, the opposite was true of our measures of perceived trustworthiness, certainty, accuracy, reliability, and trust in the tool as a whole; the selected treatment options; the numbers; and the algorithm, all of which were more reliably predicted by generalized institutional trust than by trust in the specific institution whose logo appeared (although both variables were predictive).

These are in line with trust transference theory and prior empirical work on institutional trust. Both trust in specific institutions and more generalized forms of institutional trust affect participants’ answers to survey questions;^44^ it has been hypothesised that individuals rely more on generalized institutional trust when answering questions about institutions with which they are less familiar.^45^ What is less clear is the differential role that generalized and specific institutional trust plays with respect to how individuals interpret and make decisions based on information such as quantified estimates of treatment benefits in online tools. Our finding that generalized institutional trust best predicted our key measures of trust, whereas trust in specific institutions better predicted the weight that individuals said they placed on the institutions behind the tool when making their decision, suggests a role for both factors in different aspects of the decision-making process.

For individuals who initially opted for passive treatment, those with higher specific and generalized institutional trust were more likely to switch to active treatment (partial nephrectomy) after viewing the tool, with generalized institutional trust being the stronger predictor in the 2 UK samples and trust in the specific organization the stronger predictor in the international sample. However, these variables did not predict whether participants who initially chose nephrectomy would switch to monitoring only. One possible explanation is that, despite our attempts to frame the hypothetical situation as one in which the doctor was informing the patient of options rather than persuading them to make a particular choice, the situation may have been perceived by some participants as one in which the doctor was trying to convince them to choose active treatment. In this case, participants who had more trust in medical/scientific institutions generally—or the institution associated with the tool specifically—may have been more likely to be persuaded. Previous work has found that those who are more skeptical about a topic to start with suffer more cognitive reactance when they detect a persuasive element to communications, in a direction conflicting with their prior beliefs.^46^ Alternatively, the size of the benefit communicated in the tool might have been compelling, with more trusting individuals being more likely to believe that this was indeed the size of the benefit that they could expect and less trusting individuals perhaps being wary of the estimate or considering that the risks of active treatment might be higher. In either case, the fact that these variables were consistently predictive of whether individuals switched from passive to active treatment suggests an overriding role of institutional trust in how people respond to prognostic information.

Familiarity in the cued institution was also more predictive of a wide range of variables in the international sample than in the UK sample. While ceiling effects in the UK samples may have contributed, variability in familiarity ratings was lowest in the United Kingdom (Supplementary Table S11). More research is needed to determine exactly what role familiarity plays in individuals’ reliance on generalized versus specific institutional trust.

Our findings also suggested that participants’ levels of trust in the prognostic online tool, and in how they weighted the information in their treatment decisions, were influenced by their psychological distance to the treatment scenario, as assessed by a multidimensional measure of *hypotheticality*. Participants’ feelings about whether the situation in an experiment feels authentic, plausible, and so forth are rarely used by trust researchers, and rarely adjusted for, yet we found that this measure accounted for as much variance (or more, particularly in experiment 1) as generalized institutional trust across many trust-related variables. We hope that our modification of Fishbein and Raven’s^37^ hypotheticality measure proves useful in future studies investigating the role of psychological distance on trust. In contrast, cues about the certainty or accuracy of the information itself were largely overlooked or made no difference to people’s response to the information, even when they were made explicit, for example, when they were preceded by the word “ATTENTION:” in bold font and when weaknesses were pointed out explicitly (“the tool’s estimates are less well tested for people with the characteristics you have selected”). This has ramifications for practice, which we touch on briefly in the conclusion.

### Limitations

The chief limitation of these experiments may be that the key findings were largely the result of exploratory analyses applied to exploratory and novel measures. Thankfully, several of our findings were consistent across multiple experiments, strengthening our confidence in their reproducibility; however, caution is nonetheless warranted. An additional drawback of our measures of generalized institutional trust and trust in the cued institution is that the items for the latter scale were included in the former scale, meaning that institutional trust is expected to be correlated with trust in the cued institution merely because of the way the scales were designed. This correlation would likely have been present even if the scales had not used overlapping items, as “responses to questions on trust in institutions reflect the respondent’s trust both in institutions generally as well as in the specific institution that is the focus of the question.”^44^ However, the fact that these measures were only modestly correlated (*r* = 0.61−0.73) and behaved in consistent ways across the experiments—in particular, with trust in the cued institution being a better predictor of weight on the institutions behind the tool, and generalized institutional trust being a better predictor of most other trust-related variables—provides some reassurance that they are measuring meaningfully different constructs despite their overlap.

The fact that we used UK institutions in an international sample allowed experiments 1 and 2 to use identical stimuli. However, it also meant that these experiments differed not only in terms of the sample population but also in terms of their familiarity to the UK institutions described, complicating the interpretation. Interestingly, familiarity was more predictive of trustworthiness, certainty, reliability, accuracy, and our trust variables in experiment 2 than in experiment 1. One possible explanation for this difference could be that participants in the international sample in experiment 2 who reported high familiarity with the UK institutions endorsing the tool may have in fact had actual, firsthand knowledge and experience with these institutions, whereas it is possible that UK participants in experiment 1 reported high familiarity because of the strong presence of institutions like the University of Cambridge in the public space and in UK media, even if they did not have actual firsthand experience with that specific institution. However, future work may wish to investigate this relationship further.

We have already discussed how the question we intended as an attention check was confounded with condition (being harder in the “no logo” condition than in the others) in the pilot, making the observed interactions difficult to interpret; this study also suffered from a gender imbalance, as more than 60% of participants were female. Both of these issues were addressed in experiment 1. However, all 3 samples suffered from low diversity in terms of ethnicity, confidence in using online tools and information, and lack of actual experience with the medical condition described, so even our most consistent findings may not generalize as widely as one might hope.

Relatedly, the predictive power of hypotheticality, while intriguing, also casts some doubt on the generalizability of scenario-based medical decision-making experiments to real-life contexts, including this one. That said, measuring hypotheticality enables it to be adjusted for, and we observed the same patterns of effects in the regressions conducted in this article when hypotheticality was included as a predictor. Furthermore, for some particularly important variables (e.g., propensity of participants to switch to active treatment), hypotheticality was not a significant predictor at all. It may be that scales of “trust” correlate with scales of hypotheticality partially due to semantic overlap on the scale items (e.g., “untrustworthy ⇔ trustworthy” v. “inauthentic ⇔ authentic”) and are weaker predictors of behavioral intent.

## Conclusions

First, the importance of the trust measures underscores the high importance of endorsing and supporting institutions of online tools to take actions that build rapport and trust and to demonstrate trustworthiness to the users of the tools they develop and support. An individual’s trust in the organizations that provide online tools and are represented on Web sites in institutional logos, such as the medical and scientific institutions behind the prognostic medical online tool in our experiments, are likely to be a dominant factor in how they respond to the personalized online information provided, irrespective of how that information is communicated.

Second, clinicians and communicators of online medical information may not need to be concerned about being upfront about uncertainty if their aim is to be perceived as trustworthy. Even our bold statements of uncertainty generally had little or no effect on the perceived trustworthiness in the data source or in the numbers being communicated. However, this means, that for those wanting to alert their audience to low accuracy/precision, the cues of uncertainty that we tested are not enough. To truly communicate that the evidence supporting a claim is weak enough that it is appropriate to downgrade one’s impression of its precision or accuracy, developers of online, algorithmic tools may need to go as far as describing the quality of evidence supporting an intervention explicitly as “low,” which has been shown to affect people’s perceptions of the trustworthiness of a number and induce them to be more skeptical of the potential benefit communicated.^20,21^

## Supplemental Material

sj-docx-1-mpp-10.1177_23814683241226660 – Supplemental material for What Affects Perceived Trustworthiness of Online Medical Information and Subsequent Treatment Decision Making? Click here for additional data file. Supplemental material, sj-docx-1-mpp-10.1177_23814683241226660 for What Affects Perceived Trustworthiness of Online Medical Information and Subsequent Treatment Decision Making? by Gabriel Recchia, Karin S. Moser and Alexandra L.J. Freeman in MDM Policy & Practice
