# Supplementary material for: What Affects Perceived Trustworthiness of Online Medical Information and Subsequent Treatment Decision Making? Randomized Trials on the Role of Uncertainty and Institutional Cues
Source: MDM Policy Pract. 2024 Feb 15;9(1):23814683241226660. doi: 10.1177/23814683241226660 (PMC10870812; doi:10.1177/23814683241226660)
Supplement: sj-docx-1-mpp-10.1177_23814683241226660 – Supplemental material for What Affects Perceived Trustworthiness of Online Medical Information and Subsequent Treatment Decision Making? [file sj-docx-1-mpp-10.1177_23814683241226660.docx]

## Appendix 1: Details of Pilot

## Method

### Design

The pilot used a 3 (*uncertainty cue*) × 3 (*institution cue*) between-subjects factorial design. The three uncertainty cue conditions were: no statement of uncertainty; a statement that “the true benefit to you may be a bit higher or lower than that shown because PREDICT only displays an estimated average benefit”; and a statement that “the true benefit to you may be a bit higher or lower than that shown because PREDICT is based on data from a selection of hospitals in the UK”. The institution cue conditions were no logo, the logo of Cambridge University, or the logo of the UK’s National Health Service (NHS). The pilot was preregistered at <https://osf.io/n43zm>.

**Measures**
The measures used in the pilot were nearly identical to those described in Experiment 1 of the main text, with some differences enumerated here. The pilot did not include measures of psychological distance. Furthermore, in the pilot, NHS and Cambridge were the only institutional logos used, while Experiment 1 added GlaxoSmithKline. Therefore, the pilot did not include “Yes, the logo of a pharmaceutical company” as a response option for the gist recall question “Were there any institutional logos associated with the tool?”, nor items 9 and 10 of Experiment 1’s institutional trust measure and ‘change in institutional trust’ measure. The question about how much participants associated each institution with science, teaching, or healthcare was also not present in the pilot. The pilot also used a 5-point scale for worry, while Experiment 1 used a 7-point scale. The pilot also used only the standard Berlin adaptive numeracy test (44) rather than the extended numeracy measure described in the main text. Differences between the attention check question used in the pilot and Experiment 1 are described in the main text. Finally, the two treatment options described in the opening scenario were also made more explicit in Experiment 1 by adding the phrase “These two treatment options are **monitoring only** and **partial nephrectomy**”, which was not present in the pilot. Column 1 of Table S2 illustrates the differences between the questionnaires used in each experiment.

### Participants

A power analysis conducted in G*Power indicated 1,089 participants would be required to achieve 95% power to detect an effect of size *f* = 0.15 in perceived trustworthiness at α = 0.05, assuming alpha adjustment with the Benjamini-Hochberg procedure at *k* = 5 (effectively reducing α to .01 to accommodate the test with the lowest p-value). *f* was chosen on the basis of previous work (45). 1,089 participants recruited through the Prolific^[[1]](#footnote-1)^ platform completed the experiment. The only eligibility criteria were that participants must be 18 years or older and resident in the United Kingdom. Participants were paid £2.18 for their participation. Demographics appear in Table S1.

**Results**After the 210 participants failing the attention check were removed, 879 participants remained for analysis; the proportion of participants analyzed in each condition is reported in Table 1. Perceived accuracy, reliability, certainty, and trustworthiness were mutually correlated (all pairs *r* > 0.7; Table S3). Confirmatory factor analysis indicated that a model with perceived accuracy, reliability, certainty, and trustworthiness as four separate factors achieved a better chi-squared fit index, AIC, BIC, and RMSEA than a one-factor model, but fit was poor overall (Table S4). Preregistered ANOVAs did not find main effects of uncertainty cues or institutional cues on perceived certainty, accuracy, reliability or trustworthiness. There was an interaction between uncertainty and institutional cues for perceived trustworthiness (see Table A10), which was lower among those viewing the Cambridge logo than the NHS logo, but only when no uncertainty cue was present (Figure A8). This was consistent with levels of agreement with the statement “I trust the University of Cambridge” (mean 5.496: ‘somewhat agree’, 95% CI 5.42 – 5.57) and “I trust the NHS”, which was higher (mean 5.854: ‘agree’, 95% CI 5.78 – 5.93). However, this interaction was not significant after alpha adjustment. Neither numeracy nor institutional trust moderated the relationship between institutional cues and perceived trustworthiness, but institutional trust was a significant predictor of perceived trustworthiness (*F* = 72.20, *p* < 0.001, η^2^_G_ = 0.077), as was numeracy (*F* = 9.22, *p* = 0.002, η^2^_G_ = 0.010).

**Table A10:** ANOVA results for primary and exploratory measures, pilot experiment.

|  | Uncertainty cues | | | Institutional cues | | | Uncertainty cue × institutional cue interaction | | |
| --- | --- | --- | --- | --- | --- | --- | --- | --- | --- |
|  | *F* | *p* | *η^2^_G_* | *F* | *p* | *η^2^_G_* | *F* | *p* | *η^2^_G_* |
| Primary measures |  |  |  |  |  |  |  |  |  |
| Trustworthiness | 0.14 | 0.87 | 0.000 | 2.07 | 0.13 | 0.005 | 2.83 | **0.02*** | **0.013** |
| Certainty | 0.83 | 0.44 | 0.002 | 1.33 | 0.26 | 0.003 | 0.89 | 0.47 | 0.004 |
| Accuracy | 0.43 | 0.65 | 0.001 | 0.65 | 0.52 | 0.001 | 0.86 | 0.49 | 0.004 |
| Reliability | 0.56 | 0.57 | 0.001 | 0.83 | 0.44 | 0.002 | 2.08 | 0.08 | 0.009 |
| Exploratory measures |  |  |  |  |  |  |  |  |  |
| Trust in tool as a whole | 0.79 | 0.46 | 0.002 | 1.51 | 0.22 | 0.003 | 2.61 | **0.03*** | **0.012** |
| Trust in treatments | 0.42 | 0.66 | 0.001 | 2.07 | 0.13 | 0.005 | 1.01 | 0.40 | 0.005 |
| Trust in numbers | 0.31 | 0.73 | 0.001 | 0.62 | 0.54 | 0.001 | 1.94 | 0.10 | 0.009 |
| Trust in algorithm | 0.31 | 0.73 | 0.001 | 1.17 | 0.31 | 0.003 | 0.96 | 0.43 | 0.004 |
| Weight placed on tool | 0.13 | 0.88 | 0.000 | 3.65 | **0.03*** | **0.008** | 0.54 | 0.71 | 0.002 |
| % wt on numbers | 0.51 | 0.60 | 0.001 | 5.52 | **0.004**** | **0.013** | 2.06 | 0.08 | 0.009 |
| % wt on institutions | 0.92 | 0.40 | 0.002 | 48.63 | **<.001***** | **0.101** | 0.66 | 0.62 | 0.003 |
| % wt on algorithm | 0.76 | 0.47 | 0.002 | 4.91 | **0.008**** | **0.011** | 0.90 | 0.47 | 0.004 |
| % wt on data | 1.96 | 0.14 | 0.004 | 8.30 | **<.001***** | **0.019** | 0.74 | 0.57 | 0.003 |
| % wt on other | 2.04 | 0.13 | 0.005 | 0.81 | 0.44 | 0.002 | 0.47 | 0.76 | 0.002 |
| Worry | 0.01 | 0.99 | 0.000 | 0.06 | 0.94 | 0.000 | 0.13 | 0.97 | 0.001 |
| Information sufficiency | 0.69 | 0.50 | 0.002 | 0.06 | 0.94 | 0.000 | 1.15 | 0.33 | 0.005 |
| Decision confidence | 2.12 | 0.12 | 0.005 | 0.25 | 0.78 | 0.001 | 2.18 | 0.07 | 0.010 |
| Change in decision confidence | 1.11 | 0.33 | 0.003 | 0.99 | 0.37 | 0.002 | 0.75 | 0.56 | 0.003 |

***Note.*** * = p < .05; ** = p < .01; *** = p < .001.


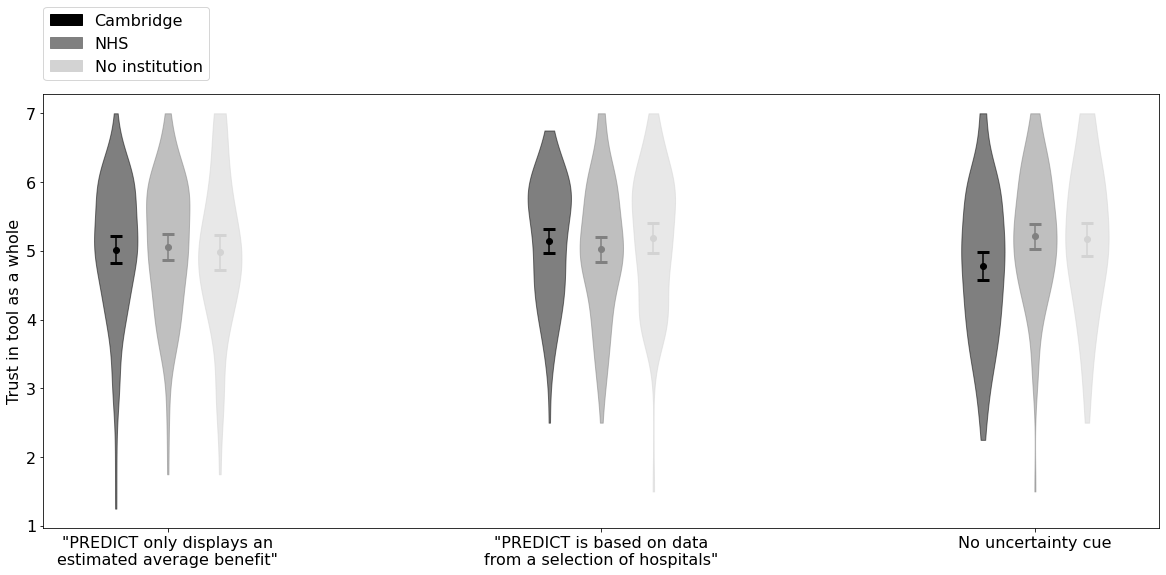


**Figure A9:** Interactions between uncertainty cue and institutional cues in the pilot. Trust in the tool as a whole and perceived trustworthiness were lowest among individuals who saw the Cambridge logo and no uncertainty cue, and were comparable for all other conditions.

**Exploratory analyses.** Analogous ANOVAs were conducted on all continuous exploratory variables; Table A10 reports the results. There was an interaction for the ANOVA with trust in the tool as the dependent variable, with this being lowest among individuals who saw the Cambridge logo and no uncertainty cue (**Figure A9**). There was a main effect of institutional cue for weight placed on the PREDICT tool, with Tukey’s post-hocs suggesting that weight was marginally lower when the Cambridge logo appeared (M = 65.0, CI: 62.2 – 67.8) vs. no logo at all (M = 70.2, CI: 66.9 – 73.4), *p_adj_* = 0.045. There were stronger effects for the *proportion of this weight* that participants reported placing on different information subtypes; the strongest effect by far was on the percentage of weight participants said they placed on institutions (η^2^_G_ = 0.101, **Figure A9**), with Tukey’s post-hocs showing that this was highest in the presence of the NHS cue, followed by Cambridge, followed by no institutional cue. Significant effects were also found for the proportion of weight participants said they placed on data (η^2^_G_ = 0.019), numbers (η^2^_G_ = 0.013), and the algorithm (η^2^_G_ = 0.011) (**Figure A10**).


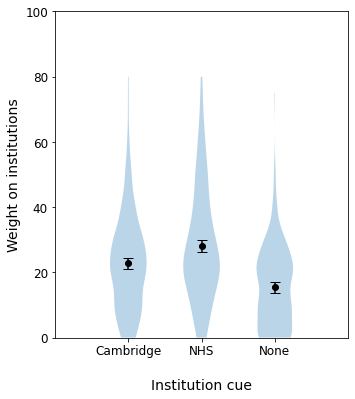


**Figure A10:** Proportion of weight that participants reported placing on “the institutions behind the development of Predict” across different institution cue conditions, pilot experiment.

**Table A11:** Coefficients and p-values for covariates in linear regression models, pilot experiment. Each model included uncertainty cue, institutional cue, and a single covariate. “Base model” refers to model with institutional and uncertainty cues only.

| Model | *Base model (all participants)* | *Base model + generalized institutional trust (all participants)* | | | *Base model (participants shown institutional cues only)* | *Base model + trust in cued institution (participants shown institutional cues only)* | | | *Base model + generalized institutional trust (participants shown institutional cues only)* | | |
| --- | --- | --- | --- | --- | --- | --- | --- | --- | --- | --- | --- |
| n | *879* | *879* | | | *645* | *645* | | | *645* | | |
|  | *Model* | *Generalized institutional trust* | | *Model* | *Model* | *Trust in cued institution* | | *Model* | *Generalized institutional trust* | | *Model* |
|  | *Adjusted r^2^* | *B* | *p* | *Adjusted r^2^* | *Adjusted r^2^* | *B* | *p* | *Adjusted r^2^* | *B* | *p* | *Adjusted r^2^* |
| **Primary measures** |  |  |  |  |  |  |  |  |  |  |  |
| Trustworthiness | .000 | 0.39 | **<.001***** | **0.077** | -.000 | 0.30 | **<.001***** | **0.075** | 0.43 | **<.001***** | **0.086** |
| Certainty | .000 | 0.30 | **<.001***** | **0.053** | -.002 | 0.24 | **<.001***** | **0.054** | 0.34 | **<.001***** | **0.058** |
| Accuracy | -.002 | 0.27 | **<.001***** | **0.050** | -.002 | 0.23 | **<.001***** | **0.065** | 0.31 | **<.001***** | **0.069** |
| Reliability | -.002 | 0.34 | **<.001***** | **0.063** | -.002 | 0.27 | **<.001***** | **0.069** | 0.38 | **<.001***** | **0.076** |
| **Exploratory measures** |  |  |  |  |  |  |  |  |  |  |  |
| Trust in tool as a whole | .000 | 0.36 | **<.001***** | **0.079** | -.000 | 0.28 | **<.001***** | **0.086** | 0.41 | **<.001***** | **0.099** |
| Trust in treatments | .001 | 0.33 | **<.001***** | **0.071** | -.002 | 0.24 | **<.001***** | **0.060** | 0.34 | **<.001***** | **0.066** |
| Trust in numbers | -.003 | 0.34 | **<.001***** | **0.059** | -.004 | 0.27 | **<.001***** | **0.064** | 0.39 | **<.001***** | **0.077** |
| Trust in algorithm | -.001 | 0.28 | **<.001***** | **0.042** | -.000 | 0.25 | **<.001***** | **0.061** | 0.33 | **<.001***** | **0.058** |
| Weight placed on tool | .004 | 2.93 | **0.005**** | **0.012** | .004 | 1.39 | 0.15 | 0.005 | 3.25 | **0.01*** | **0.012** |
| % wt on numbers | .009 | -0.02 | 0.98 | 0.008 | .000 | -1.11 | 0.12 | 0.002 | -0.15 | 0.88 | -0.002 |
| % wt on institutions | .097 | 1.66 | **0.01*** | **0.103** | .023 | 2.44 | **<.001***** | **0.046** | 2.00 | **0.02*** | **0.031** |
| % wt on algorithm | .009 | -0.79 | 0.09 | 0.011 | .003 | -0.44 | 0.28 | 0.004 | -0.40 | 0.46 | 0.003 |
| % wt on data | .018 | -0.68 | 0.28 | 0.018 | .003 | -0.69 | 0.21 | 0.004 | -1.51 | **0.04*** | **0.008** |
| % wt on other | .003 | -0.17 | 0.73 | 0.002 | .004 | -0.21 | 0.66 | 0.003 | 0.05 | 0.93 | 0.003 |
| Worry | -.004 | -0.22 | **<.001***** | **0.048** | -.005 | -0.13 | **<.001***** | **0.028** | -0.23 | **<.001***** | **0.054** |
| Information sufficiency | -.003 | 0.38 | **<.001***** | **0.027** | -.004 | 0.28 | **<.001***** | **0.022** | 0.42 | **<.001***** | **0.030** |
| Decision confidence | -.001 | 0.21 | **<.001***** | **0.036** | -.003 | 0.11 | **<.001***** | **0.012** | 0.21 | **<.001***** | **0.029** |
| Change in decision confidence | -.000 | 0.03 | 0.45 | -0.001 | -.002 | 0.02 | 0.46 | -0.002 | 0.02 | 0.71 | -0.003 |

Notably, covariates had far stronger effects on the primary measures than the manipulated variables (Table A11). As the ‘trust in the cued institution’ measure was constructed from items in the generalized institutional trust measure, these were correlated (*r =* 0.73) and accounted for comparable amounts of variance on most measures (Table A11); analyses including both covariates in the same model are reported in Table S5. The logistic regressions likewise did not show significant effects of uncertainty cues or institution cues, but generalized institutional trust and trust in the cued institution predicted the propensity to switch to active treatment for those participants who had initially opted for passive treatment before seeing the tool (Table A12, Figure A9, Figure A10). Additional exploratory results are reported elsewhere in the supplementary materials.

**Table A12:** Coefficients and p-values for covariates in logistic regression models including uncertainty cue, institutional cue, and a single covariate for exploratory binary measures, pilot experiment. McFadden’s pseudo-R^2^s (43) are simply one minus the ratio of the model’s log-likelihood to that of a model with intercept only, and reflect the variance accounted for by the logistic regression model.

|  | Model | | | | | |
| --- | --- | --- | --- | --- | --- | --- |
|  | Inst., unc. cues only | | Inst., unc. cues + generalized  institutional trust | | | |
|  |  |  |  |  | *Generalized institutional trust* | |
| **Dependent variable** | *pseudo r^2^* | | *pseudo r^2^* | | *B* | *p* |
| Switched their choice after viewing tool  (Switched = 1)  N = 879 | 0.004 | | 0.004 | | 0.07 | 0.48 |
| Switched to active treatment after viewing tool (Switched = 1) (Only those who initially chose passive treatment: N = 293) | 0.007 | | 0.021 | | **0.32** | **0.03*** |
| Switched to passive treatment after viewing tool (Switched = 1)  (Only those who initially chose active treatment: N = 586) | 0.003 | | 0.003 | | -.07 | 0.63 |

**Table A13:** Pilot experiment: Coefficients and p-values for covariates in logistic regression models including uncertainty cue, institutional cue, and a single covariate for exploratory binary measures, restricted to participants who were shown an institutional cue. McFadden’s pseudo-R^2^s (43) are simply one minus the ratio of the model’s log-likelihood to that of a model with intercept only, and reflect the variance accounted for by the logistic regression model.

|  | Model | | | | | |  |  |  |  |
| --- | --- | --- | --- | --- | --- | --- | --- | --- | --- | --- |
|  | Inst., unc. cues only | | Inst., unc. cues + trust in cued institution | | | | Inst., unc. cues + generalized institutional trust | | | |
|  |  |  |  |  | *Trust in cued institution* | |  |  | *Generalized institutional trust* | |
| **Dependent variable** | *pseudo r^2^* | | *pseudo r^2^* | | *B* | *p* | *pseudo r^2^* | | *B* | *p* |
| Switched their choice after viewing tool  (Switched = 1)  (Only those who were shown an institutional cue: N = 645) | 0.007 | | 0.011 | | 0.16 | 0.09 | 0.009 | | 0.13 | 0.28 |
| Switched to active treatment after viewing tool (Switched = 1) (Only those who initially chose passive treatment and were shown an institutional cue: N = 209) | 0.010 | | 0.027 | | **0.30** | **0.04*** | 0.036 | | **0.47** | **0.01*** |
| Switched to passive treatment after viewing tool (Switched = 1)  (Only those who initially chose active treatment and were shown an institutional cue: N = 436) | 0.006 | | 0.008 | | 0.10 | 0.40 | 0.007 | | -0.05 | 0.78 |

##
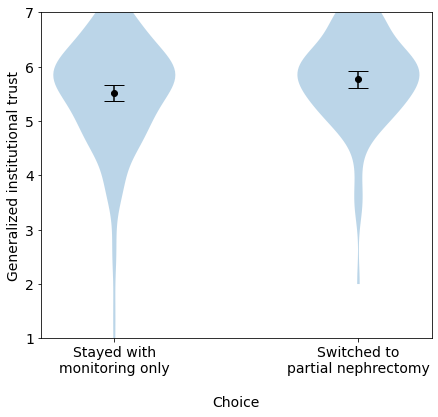


**Figure A11:** Differences in generalized institutional trust between participants who stayed with monitoring only vs. those who switched to partial nephrectomy, among participants who initially chose monitoring only, pilot experiment.

## 2.4. Discussion

Given that our sample’s trust in Cambridge University was lower that their trust in the NHS, the interactions we observed suggested an intriguing hypothesis: that institutional cues only exert an influence on trust when uncertainty cues were absent. Unfortunately, participants’ likelihood of remembering the institutional cue (passing the attention check) differed based on which institutional cue condition they were in, introducing possible bias. If remembering that the tool contained the Cambridge logo required less attentiveness on average than remembering that it contained no institutional logo at all, excluding participants who failed the check could have resulted in differences in the attentiveness of participants between conditions. We therefore attempted to replicate these interactions in another study on a UK population (Experiment 1) and an international population (Experiment 2), both of which used an alternative attention check. The interactions did not replicate.

Institutional cues predicted the overall amount of weight that participants said they placed on the tool when making their decision, and had a particularly strong effect on the proportion of this weight that people said they placed on the institutions behind the development of the tool (*η^2^_G_* = 0.10). Furthermore, in contrast to the small amount of variance accounted for by institutional and uncertainty cues, *generalized institutional trust* and *trust in the cued institution* also each explained much more variation in trust-related variables (Table A11).

## Supplementary Information

Table S1. Characteristics of participants participating in the experiments.

|  | Pilot | | Experiment 1 | | | Experiment 2 | | |
| --- | --- | --- | --- | --- | --- | --- | --- | --- |
|  | Total N (%) | Analysed N (%) | Total N (%) | Analysed N (%) | | Total N (%) | Analysed N (%) | |
| **Gender** |  |  |  |  | |  | |  |
| Female | 680 (62%) | 541 (62%) | 929 (50%) | | 904 (51%) | 988 (45%) | | 935 (45%) |
| Male | 398 (37%) | 328 (37%) | 898 (49%) | | 855 (48%) | 1164 (53%) | | 1101 (53%) |
| Other (please specify) |  |  |  | |  |  | |  |
| Agender | 1 (<1%) | 1 (<1%) | 0 (0%) | | 0 (0%) | 3 (<1%) | | 3 (<1%) |
| Gender non-conforming | 0 (0%) | 0 (0%) | 1 (<1%) | | 1 (<1%) | 3 (<1%) | | 3 (<1%) |
| Genderqueer | 0 (0%) | 0 (0%) | 2 (<1%) | | 2 (<1%) | 0 (0%) | | 0 (0%) |
| Nonbinary | 4 (<1%) | 3 (<1%) | 7 (<1%) | | 7 (<1%) | 13 (1%) | | 13 (1%) |
| Missing or Prefer not to say | 6 (1%) | 6 (1%) | 8 (<1%) | | 8 (<1%) | 14 (1%) | | 13 (1%) |
| **Age group** |  |  |  | |  |  | |  |
| 18-34 years | 617 (57%) | 522 (59%) | 1027 (56%) | | 997 (56%) | 1816 (83%) | | 1718 (83%) |
| 35-54 years | 349 (32%) | 267 (30%) | 638 (35%) | | 609 (34%) | 319 (15%) | | 305 (15%) |
| 55+ years | 118 (11%) | 86 (10%) | 171 (9%) | | 163 (9%) | 43 (2%) | | 39 (2%) |
| Missing or Prefer not to say | 4 (<1%) | 4 (<1%) | 9 (<1%) | | 8 (<1%) | 7 (<1%) | | 6 (<1%) |
| **Education** |  |  |  | |  |  | |  |
| Below upper secondary | 181 (17%) | 144 (16%) | 237 (13%) | | 226 (13%) | 284 (13%) | | 265 (14%) |
| Upper secondary | 380 (35%) | 315 (36%) | 590 (32%) | | 573 (32%) | 629 (29%) | | 593 (30%) |
| Above upper secondary  (HND or other degree) | 518 (48%) | 410 (47%) | 1008 (55%) | | 968 (54%) | 1142 (52%) | | 1085 (55%) |
| Missing or Prefer not to say | 10 (1%) | 10 (1%) | 10 (1%) | | 10 (1%) | 130 (6%) | | 12 (1%) |
| **Ethnicity** |  |  |  | |  |  | |  |
| Asian/Asian British | 79 (7%) | 67 (8%) | 150 (8%) | | 141 (8%) | 69 (3%) | | 63 (3%) |
| Black/African/Caribbean/Black British | 38 (3%) | 31 (4%) | 52 (3%) | | 50 (3%) | 70 (3%) | | 61 (3%) |
| Mixed/Multiple ethnic groups | 41 (4%) | 35 (4%) | 54 (3%) | | 52 (3%) | 65 (3%) | | 63 (3%) |
| White | 919 (84%) | 734 (84%) | 1553 (84%) | | 1502 (85%) | 1926 (88%) | | 1828 (88%) |
| Other ethnic group | 6 (1%) | 6 (1%) | 13 (1%) | | 11 (1%) | 37 (2%) | | 35 (2%) |
| Missing or Prefer not to say | 6 (1%) | 6 (1%) | 23 (1%) | | 21 (1%) | 18 (1%) | | 18 (1%) |
| **Direct experience with cancer?** |  |  |  | |  |  | |  |
| Yes, personally | 36 (3%) | 29 (3%) | 57 (3%) | | 55 (3%) | 34 (2%) | | 32 (2%) |
| Yes, someone close to me | 574 (53%) | 458 (52%) | 1046 (57%) | | 1009 (57%) | 1063 (49%) | | 1011 (49%) |
| No | 463 (43%) | 377 (43%) | 711 (39%) | | 683 (38%) | 1061 (49%) | | 998 (48%) |
| Missing or Prefer not to say | 16 (1%) | 15 (2%) | 31 (2%) | | 30 (2%) | 27 (1%) | | 27 (1%) |
| **Direct experience with kidney cancer?** |  |  |  | |  |  | |  |
| Yes, personally | 2 (<1%) | 1 (<1%) | 4 (<1%) | | 4 (<1%) | 0 (0%) | | 0 (0%) |
| Yes, someone close to me | 62 (6%) | 47 (5%) | 91 (5%) | | 88 (5%) | 117 (5%) | | 109 (5%) |
| No | 1020 (94%) | 826 (94%) | 1730 (94%) | | 1666 (94%) | 2053 (94%) | | 1944 (94%) |
| Missing or Prefer not to say | 5 (<1%) | 5 (1%) | 20 (1%) | | 19 (1%) | 15 (1%) | | 15 (1%) |
| **Prior experience with PREDICT?** |  |  |  | |  |  | |  |
| Yes | 16 (1%) | 12 (1%) | 9 (<1%) | | 8 (<1%) | 119 (5%) | | 110 (5%) |
| No | 1065 (98%) | 861 (98%) | 1825 (99%) | | 1758 (99%) | 2035 (93%) | | 1929 (93%) |
| Missing or Prefer not to say | 8 (1%) | 6 (1%) | 11 (1%) | | 11 (1%) | 31 (1%) | | 29 (1%) |
| **Numeracy (Berlin Numeracy Score)** |  |  |  | |  |  | |  |
| 1 (low) | 323 (30%) | 254 (29%) | 507 (27%) | | 481 (27%) | 627 (29%) | | 572 (28%) |
| 2 (moderately low) | 388 (36%) | 312 (35%) | 643 (35%) | | 621 (35%) | 634 (29%) | | 616 (30%) |
| 3 (moderately high) | 133 (12%) | 109 (12%) | 240 (13%) | | 235 (13%) | 314 (14%) | | 297 (14%) |
| 4 (high) | 245 (22%) | 204 (23%) | 455 (25%) | | 440 (25%) | 610 (28%) | | 583 (28%) |
| **Numeracy (Combined score)** |  |  |  | |  |  | |  |
| 1 (lowest) | N/A (not recorded) | | 8 (<1%) | | 7 (<1%) | 25 (1%) | | 16 (1%) |
| 2 |  |  | 55 (3%) | | 49 (3%) | 68 (3%) | | 63 (3%) |
| 3 |  |  | 172 (9%) | | 165 (9%) | 195 (9%) | | 177 (9%) |
| 4 |  |  | 264 (14%) | | 253 (14%) | 287 (13%) | | 266 (13%) |
| 5 |  |  | 327 (18%) | | 317 (18%) | 379 (17%) | | 367 (18%) |
| 6 |  |  | 445 (24%) | | 430 (24%) | 472 (22%) | | 453 (22%) |
| 7 |  |  | 200 (11%) | | 193 (11%) | 273 (12%) | | 258 (12%) |
| 8 (highest) |  |  | 374 (20%) | | 363 (20%) | 486 (22%) | | 468 (23%) |
| **Native language** |  |  |  | |  |  | |  |
| English | 1059 (97%) | 853 (97%) | 1636 (89%) | | 1578 (89%) | 386 (18%) | | 365 (18%) |
| Other | 27 (2%) | 23 (3%) | 186 (10%) | | 176 (10%) | 1757 (80%) | | 1661 (80%) |
| Missing or Prefer not to say | 3 (<1%) | 3 (<1%) | 23 (1%) | | 23 (1%) | 42 (2%) | | 42 (2%) |
| **If English is not your native language, what is your level of English?** |  |  |  | |  |  | |  |
| Beginner | 0 (0%) | 0 (0%) | 1 (1%) | | 1 (1%) | 22 (1%) | | 16 (1%) |
| Intermediate | 9 (35%) | 8 (36%) | 14 (8%) | | 14 (8%) | 629 (29%) | | 599 (29%) |
| Advanced | 17 (65%) | 14 (64%) | 167 (92%) | | 157 (91%) | 1097 (50%) | | 1039 (50%) |
| **Were you born in the UK?** |  |  |  | |  |  | |  |
| Yes | 1009 (93%) | 809 (92%) | 1482 (80%) | | 1435 (85%) | 204 (9%) | | 196 (9%) |
| No | 73 (7%) | 64 (7%) | 349 (19%) | | 238 (14%) | 1928 (88%) | | 1821 (88%) |
| Missing or Prefer not to say | 7 (1%) | 6 (1%) | 14 (1%) | | 14 (1%) | 53 (2%) | | 51 (2%) |
| **How confident are you using online tools and information on the internet?** |  |  |  | |  |  | |  |
| 1 (Not confident at all) | 1 (<1%) | 0 (0%) | 1 (<1%) | | 1 (<1%) | 5 (<1%) | | 5 (<1%) |
| 2 | 5 (<1%) | 4 (<1%) | 4 (<1%) | | 4 (<1%) | 18 (1%) | | 17 (1%) |
| 3 | 11 (1%) | 8 (1%) | 17 (1%) | | 17 (1%) | 52 (2%) | | 49 (2%) |
| 4 | 38 (3%) | 29 (3%) | 47 (3%) | | 45 (3%) | 151 (7%) | | 142 (7%) |
| 5 | 152 (14%) | 117 (13%) | 217 (12%) | | 204 (11%) | 479 (22%) | | 446 (22%) |
| 6 | 306 (28%) | 247 (28%) | 481 (26%) | | 464 (26%) | 636 (29%) | | 605 (29%) |
| 7 (Very confident) | 576 (53%) | 474 (54%) | 1078 (58%) | | 1042 (59%) | 844 (39%) | | 804 (39%) |
| **Decision Making Preference Questionnaire – who should make treatment decisions?** |  |  |  | |  |  | |  |
| Doctor | 43 (4%) | 30 (3%) | 51 (3%) | | 50 (3%) | 101 (5%) | | 94 (5%) |
| Doctor, but should strongly consider patient’s opinion | 178 (16%) | 142 (16%) | 314 (17%) | | 300 (17%) | 415 (19%) | | 400 (19%) |
| Doctor and patient should make decisions on equal basis | 375 (34%) | 306 (35%) | 563 (31%) | | 544 (31%) | 641 (29%) | | 601 (29%) |
| Patient, but should strongly consider doctor’s opinion | 453 (42%) | 366 (42%) | 837 (45%) | | 805 (45%) | 960 (44%) | | 907 (44%) |
| Patient | 40 (4%) | 35 (4%) | 80 (4%) | | 78 (4%) | 68 (3%) | | 66 (3%) |

Table S2. Instrument

| ***Experim.*** | ***Survey prompt or measure*** |
| --- | --- |
| Pilot, 1, 2 | In this study, we will ask you to evaluate information about treatment options for kidney cancer. On the next few pages, we will first provide some information about what kidney cancer is and about a fictional tool for communicating treatment options to patients.    Please read the information carefully.  [PAGE BREAK]  **Note: You will be asked to imagine yourself in a fictional medical scenario.** This is a realistic scenario in that the treatment options and the decision options are true.  However, the numbers you will see are fictional: they are not the real-world statistics for people who have these treatments in real life.  [PAGE BREAK] |
| Pilot, 1, 2 | **What is kidney cancer?**  Kidney cancer, also called renal cancer, is one of the most common types of cancer in the UK.    It usually affects adults in their 60s or 70s and is rare in people under 50. It can often be cured if it's caught early. But a cure probably won't be possible if it's not diagnosed until after it has spread beyond the kidney.    There are several types of kidney cancer. The most common type is called renal cell carcinoma. **Localised renal cell carcinoma** is cancer that is inside the kidney and hasn’t spread to other parts of the body.  **Please confirm that you have carefully read this information:**  ◯ **Yes**  [PAGE BREAK] |
| Pilot, 1, 2 | **Now, please imagine you are in the following situation. Please remember that if you feel uncomfortable, you can stop participating in this study at any time.**    **Imagine that you are a person who has recently received a diagnosis of localised renal cell carcinoma (kidney cancer).**      Your doctor tells you the following:    “The outlook for kidney cancer largely depends on how big the tumour is and how far it has spread by the time it's diagnosed.    If the cancer is still small and hasn't spread beyond the kidney, surgery can often cure it. Some small, slow growing cancers may not need treatment at first.    Your cancer is small and slow-growing, and it doesn’t seem to have spread. Therefore, there are two treatment options which I would consider reasonable. What’s best for you depends on how you personally weigh up the risks and benefits of the different treatments. |
| 1, 2 | These two treatment options are **monitoring only** and **partial nephrectomy**.”  [PAGE BREAK] |
| Pilot, 1, 2 | **Monitoring only** means that the kidney cancer will be monitored, for example through regular tests to check on the cancer. If you choose **monitoring only**, you won’t have any treatment unless these tests show that the cancer may be growing more quickly or you decide you want treatment later, so you’ll avoid or delay the side effects of surgery or other treatments.  **Partial nephrectomy** refers to an operation to **remove part of the kidney** containing the cancer. For people with cancers similar to yours, this cures the cancer in most cases. However, in some cases, it does not. In addition, surgery can come with complications, such as infection, bleeding requiring blood transfusion, or post-surgical pneumonia.    **Please confirm that you have carefully read this information:**  ◯ **Yes**  [PAGE BREAK] |
| Pilot, 1, 2 | **If you had to make a choice with only the information you have learned up to now, what would you choose?**  ◯ Monitoring only  ◯ Partial nephrectomy (removing part of the kidney)  **Please rate how you feel below.**   \|  \| No, not at all 1 \| 2 \| 3 \| 4 \| Yes, extremely 5 \| \| --- \| --- \| --- \| --- \| --- \| --- \| \| Do you feel confident about the choice you made above? \| ◯ \| ◯ \| ◯ \| ◯ \| ◯ \| \| Did you feel clear about the best choice for you? \| ◯ \| ◯ \| ◯ \| ◯ \| ◯ \| \| Did you feel sure about what to choose? \| ◯ \| ◯ \| ◯ \| ◯ \| ◯ \| \| [PAGE BREAK] \| \|  \|  \|  \|  \| |
| Pilot, 1, 2 | Now, imagine that the doctor continues as follows:    “I would like to show you an online tool that some patients find helpful when deciding together with their clinicians which treatments to have. The results in this tool are produced by a computer algorithm, which is based on data from thousands of people who have been diagnosed with kidney cancer in England.    This tool can’t tell what will happen to you. But it can show you how many people with similar characteristics to you (e.g. similar tumour types, similar age, etc.) survived at least 10 years after kidney cancer diagnosis. It has information about two groups of people with similar characteristics to you:    ·      people whose cancer was monitored only  ·      people who had partial nephrectomy (removing part of the kidney)    In other words, the differences you are about to see are differences between groups of people who all had similar characteristics to you, but who chose different treatments.”  **Please confirm that you have carefully read this information:**  ◯ **Yes**  [PAGE BREAK] |
| Pilot, 1, 2 | Please take your time to look at these results. We will ask you some questions about this afterwards.  [STIMULUS]  [PAGE BREAK] |
| 1, 2 | If you would prefer not to answer this question you may leave it blank.  **Have you ever had to weigh up options in real life in a way that is at all similar to the situation presented in this study?**   \| Not at all 1 \| 2 \| 3 \| 4 \| 5 \| 6 \| Very much 7 \| \| --- \| --- \| --- \| --- \| --- \| --- \| --- \| \| ◯  [PAGE BREAK] \| ◯ \| ◯ \| ◯ \| ◯ \| ◯ \| ◯ \| |
| 1, 2 | **Please rate how the situation in this experiment felt to you along each scale below.**   \| Extremely realistic 1 \| 2 \| 3 \| 4 \| 5 \| 6 \| Extremely unrealistic 7 \| \| --- \| --- \| --- \| --- \| --- \| --- \| --- \| \| ◯ \| ◯ \| ◯ \| ◯ \| ◯ \| ◯ \| ◯ \| \| Extremely authentic 1 \| 2 \| 3 \| 4 \| 5 \| 6 \| Extremely fake 7 \| \| ◯ \| ◯ \| ◯ \| ◯ \| ◯ \| ◯ \| ◯ \| \| Extremely fictional 1 \| 2 \| 3 \| 4 \| 5 \| 6 \| Extremely real-world 7 \| \| ◯ \| ◯ \| ◯ \| ◯ \| ◯ \| ◯ \| ◯ \| \| Extremely implausible 1 \| 2 \| 3 \| 4 \| 5 \| 6 \| Extremely plausible 7 \| \| ◯ \| ◯ \| ◯ \| ◯ \| ◯ \| ◯ \| ◯ \| \| Extremely false 1 \| 2 \| 3 \| 4 \| 5 \| 6 \| Extremely true 7 \| \| ◯ \| ◯ \| ◯ \| ◯ \| ◯ \| ◯ \| ◯ \| \| Extremely impossible 1 \| 2 \| 3 \| 4 \| 5 \| 6 \| Extremely possible 7 \| \| ◯ \| ◯ \| ◯ \| ◯ \| ◯ \| ◯ \| ◯ \| \| Extremely existent 1 \| 2 \| 3 \| 4 \| 5 \| 6 \| Extremely nonexistent 7 \| \| ◯ \| ◯ \| ◯ \| ◯ \| ◯ \| ◯ \| ◯ \| \| Extremely probable 1 \| 2 \| 3 \| 4 \| 5 \| 6 \| Extremely improbable 7 \| \| ◯ \| ◯ \| ◯ \| ◯ \| ◯ \| ◯ \| ◯ \| \| Extremely unlikely 1 \| 2 \| 3 \| 4 \| 5 \| 6 \| Extremely likely 7 \| \| ◯  [PAGE BREAK] \| ◯ \| ◯ \| ◯ \| ◯ \| ◯ \| ◯ \| |
| Pilot, 1, 2 | **Please think about the results from the PREDICT: KIDNEY tool and answer the following questions.** |
| 1, 2 | **How worried would we be if you didn't pay attention? To check that you are paying attention, please select 'somewhat agree' below.**  ◯ Disagree  ◯ Somewhat disagree  ◯ Neither disagree nor agree  ◯ Somewhat agree  ◯ Agree  ◯ Completely agree |
| Pilot | **Were there any institutional logos associated with the tool?**  ◯ Yes, the logo of a healthcare institution  ◯ Yes, the logo of a university  ◯ No  ◯ Other (please specify) _________________ |
| 1, 2 | **Were there any institutional logos associated with the tool?**  ◯ Yes, the logo of a pharmaceutical company  ◯ Yes, the logo of a university  ◯ Yes, the logo of a healthcare institution  ◯ No  ◯ Other (please specify) _________________ |
| Pilot, 1, 2 | **According to the PREDICT: KIDNEY tool, which of the treatment options gives the highest percentage survival at least 10 years after diagnosis of kidney cancer?**  ◯ Monitoring only  ◯ Partial nephrectomy (removing part of the kidney)  ◯ I don’t know |
| Pilot, 1, 2 | **What percentage of people who received ‘monitoring only’ survived at least 10 years after diagnosis?**    **Please answer what you remember.**  [Slider ranging from 0 to 100] |
| Pilot, 1, 2 | **What percentage of people who received ‘partial nephrectomy’ survived at least 10 years after diagnosis?**    **Please answer what you remember.**  [Slider ranging from 0 to 100]  [PAGE BREAK] |
| Pilot, 1, 2 | In the next section, you will be asked four blocks of questions about different aspects of the “PREDICT: KIDNEY” tool: how accurate they seemed to you, how certain they seemed to you, how reliable they seemed to you, and how trustworthy they seemed to you.    Please pay close attention to the questions you are asked and answer accordingly.  [PAGE BREAK]  Please state how **accurate** these aspects of the PREDICT: KIDNEY tool seemed to you.   \|  \| Not at all **accurate** 1 \| 2 \| 3 \| 4 \| 5 \| 6 \| Very **accurate** 7 \| \| --- \| --- \| --- \| --- \| --- \| --- \| --- \| --- \| \| The Predict tool as a whole \| ◯ \| ◯ \| ◯ \| ◯ \| ◯ \| ◯ \| ◯ \| \| The selected treatment options in Predict \| ◯ \| ◯ \| ◯ \| ◯ \| ◯ \| ◯ \| ◯ \| \| The numbers in Predict \| ◯ \| ◯ \| ◯ \| ◯ \| ◯ \| ◯ \| ◯ \| \| The computer algorithm in Predict \| ◯ \| ◯ \| ◯ \| ◯ \| ◯ \| ◯ \| ◯ \| \| [PAGE BREAK] \| \|  \|  \|  \|  \|  \|  \| |

| Pilot, 1, 2 | Please state how **certain** these aspects of the PREDICT: KIDNEY tool seemed to you.   \|  \| Not at all **certain** 1 \| 2 \| 3 \| 4 \| 5 \| 6 \| Very **certain** 7 \| \| --- \| --- \| --- \| --- \| --- \| --- \| --- \| --- \| \| The Predict tool as a whole \| ◯ \| ◯ \| ◯ \| ◯ \| ◯ \| ◯ \| ◯ \| \| The selected treatment options in Predict \| ◯ \| ◯ \| ◯ \| ◯ \| ◯ \| ◯ \| ◯ \| \| The numbers in Predict \| ◯ \| ◯ \| ◯ \| ◯ \| ◯ \| ◯ \| ◯ \| \| The computer algorithm in Predict \| ◯ \| ◯ \| ◯ \| ◯ \| ◯ \| ◯ \| ◯ \| \| [PAGE BREAK] \| \|  \|  \|  \|  \|  \|  \| |
| --- | --- | --- | --- | --- | --- | --- | --- | --- | --- | --- | --- | --- | --- | --- | --- | --- | --- | --- | --- | --- | --- | --- | --- | --- | --- | --- | --- | --- | --- | --- | --- | --- | --- | --- | --- | --- | --- | --- | --- | --- | --- | --- | --- | --- | --- | --- | --- | --- | --- |

| Pilot, 1, 2 | Please state how **reliable** these aspects of the PREDICT: KIDNEY tool seemed to you.   \|  \| Not at all **reliable** 1 \| 2 \| 3 \| 4 \| 5 \| 6 \| Very **reliable** 7 \| \| --- \| --- \| --- \| --- \| --- \| --- \| --- \| --- \| \| The Predict tool as a whole \| ◯ \| ◯ \| ◯ \| ◯ \| ◯ \| ◯ \| ◯ \| \| The selected treatment options in Predict \| ◯ \| ◯ \| ◯ \| ◯ \| ◯ \| ◯ \| ◯ \| \| The numbers in Predict \| ◯ \| ◯ \| ◯ \| ◯ \| ◯ \| ◯ \| ◯ \| \| The computer algorithm in Predict \| ◯ \| ◯ \| ◯ \| ◯ \| ◯ \| ◯ \| ◯ \| \| [PAGE BREAK] \| \|  \|  \|  \|  \|  \|  \| |
| --- | --- | --- | --- | --- | --- | --- | --- | --- | --- | --- | --- | --- | --- | --- | --- | --- | --- | --- | --- | --- | --- | --- | --- | --- | --- | --- | --- | --- | --- | --- | --- | --- | --- | --- | --- | --- | --- | --- | --- | --- | --- | --- | --- | --- | --- | --- | --- | --- | --- |

| Pilot, 1, 2 | Please state how **trustworthy** these aspects of the PREDICT: KIDNEY tool seemed to you.   \|  \| Not at all **trustworthy** 1 \| 2 \| 3 \| 4 \| 5 \| 6 \| Very **trustworthy** 7 \| \| --- \| --- \| --- \| --- \| --- \| --- \| --- \| --- \| \| The Predict tool as a whole \| ◯ \| ◯ \| ◯ \| ◯ \| ◯ \| ◯ \| ◯ \| \| The selected treatment options in Predict \| ◯ \| ◯ \| ◯ \| ◯ \| ◯ \| ◯ \| ◯ \| \| The numbers in Predict \| ◯ \| ◯ \| ◯ \| ◯ \| ◯ \| ◯ \| ◯ \| \| The computer algorithm in Predict \| ◯ \| ◯ \| ◯ \| ◯ \| ◯ \| ◯ \| ◯ \| \| [PAGE BREAK] \| \|  \|  \|  \|  \|  \|  \| |
| --- | --- | --- | --- | --- | --- | --- | --- | --- | --- | --- | --- | --- | --- | --- | --- | --- | --- | --- | --- | --- | --- | --- | --- | --- | --- | --- | --- | --- | --- | --- | --- | --- | --- | --- | --- | --- | --- | --- | --- | --- | --- | --- | --- | --- | --- | --- | --- | --- | --- |
| Pilot, 1, 2 | **If you had to make a choice with only the information you have learned up to now, what would you choose?**  ◯ Monitoring only  ◯ Partial nephrectomy (removing part of the kidney)  **Please rate how you feel below.**   \|  \| No, not at all 1 \| 2 \| 3 \| 4 \| Yes, extremely 5 \| \| --- \| --- \| --- \| --- \| --- \| --- \| \| Do you feel confident about the choice you made above? \| ◯ \| ◯ \| ◯ \| ◯ \| ◯ \| \| Did you feel clear about the best choice for you? \| ◯ \| ◯ \| ◯ \| ◯ \| ◯ \| \| Did you feel sure about what to choose? \| ◯ \| ◯ \| ◯ \| ◯ \| ◯ \| \| [PAGE BREAK] \| \|  \|  \|  \|  \| |
| Pilot, 1, 2 | **Did you feel you had all of the information you needed to make a decision?**   \| No, definitely not 1 \| 2 \| 3 \| 4 \| 5 \| 6 \| Yes, definitely 7 \| \| --- \| --- \| --- \| --- \| --- \| --- \| --- \| \| ◯ \| ◯ \| ◯ \| ◯ \| ◯ \| ◯ \| ◯ \|   **When making your decision, how much weight did you put on the information provided by Predict?**  Please indicate this with a number ranging from 0 to 100. A score of 0 indicates that your decision had nothing to do with the information provided by Predict, and was entirely based on other factors. A score of 100 indicates that your decision was entirely based on the information provided by Predict, and was not influenced by other factors.  [Slider ranging from 0 to 100]  **Factors other than Predict that were relevant to your treatment decision (if any):**  [free text]  [PAGE BREAK] |
| Pilot, 1, 2 | **For this question, please focus on the information that was provided by the Predict tool.**  How much weight did you put on each type of information provided by Predict?  Type your answers as a percent out of 100. All of your answers should add up to 100.  My confidence in the numbers provided by Predict .....% : _______  The institutions behind the development of Predict .....% : _______  The algorithm that Predict is based on .....% : _______  The data the Predict algorithm is based on .....% : _______  Other: please type in the text box at the bottom of this page .....% : _______  **Other information provided by Predict that was relevant to your treatment decision:**  (free text)  [PAGE BREAK] |
| Pilot, 1, 2 | **If your opinion of the different treatment options changed once you saw the data from PREDICT: KIDNEY, can you describe how it changed? For example, you might say that you felt better about one of the treatments, worse about one of the treatments, or that your opinion did not change at all.**  (free text)  **If your opinion of the different treatment options changed once you saw the data from PREDICT: KIDNEY, can you describe why it changed?**  (free text)  [PAGE BREAK] |
| Pilot | **If you received these results, to what extent would you feel worried about your treatment options for kidney cancer?**   \| Not at all 1 \| 2 \| 3 \| 4 \| Extremely  5 \| \| --- \| --- \| --- \| --- \| --- \| \| ◯ \| ◯ \| ◯ \| ◯ \| ◯ \|   **If you received these results, to what extent would you feel anxious about your treatment options for kidney cancer?**   \| Not at all 1 \| 2 \| 3 \| 4 \| Extremely  5 \| \| \| --- \| --- \| --- \| --- \| --- \| --- \| \| ◯ \| ◯ \| ◯ \| ◯ \| ◯ \|  \|   **If you received these results, to what extent would you feel confident about your treatment options for kidney cancer?**   \| Not at all 1 \| 2 \| 3 \| 4 \| Extremely  5 \|  \|  \| \| --- \| --- \| --- \| --- \| --- \| --- \| --- \| \| ◯ \| ◯ \| ◯ \| ◯ \| ◯ \|  \|  \|   **If you received these results, to what extent would you feel good about your treatment options for kidney cancer?**   \| Not at all 1 \| 2 \| 3 \| 4 \| Extremely  5 \|  \|  \| \| --- \| --- \| --- \| --- \| --- \| --- \| --- \| \| ◯ \| ◯ \| ◯ \| ◯ \| ◯ \|  \|  \| |
| 1, 2 | **If you received these results, to what extent would you feel worried about your treatment options for kidney cancer?**   \| Not at all 1 \| 2 \| 3 \| 4 \| 5 \| 6 \| Extremely 7 \| \| --- \| --- \| --- \| --- \| --- \| --- \| --- \| \| ◯ \| ◯ \| ◯ \| ◯ \| ◯ \| ◯ \| ◯ \|   **If you received these results, to what extent would you feel anxious about your treatment options for kidney cancer?**   \| Not at all 1 \| 2 \| 3 \| 4 \| 5 \| 6 \| Extremely 7 \| \| --- \| --- \| --- \| --- \| --- \| --- \| --- \| \| ◯ \| ◯ \| ◯ \| ◯ \| ◯ \| ◯ \| ◯ \|   **If you received these results, to what extent would you feel confident about your treatment options for kidney cancer?**   \| Not at all 1 \| 2 \| 3 \| 4 \| 5 \| 6 \| Extremely 7 \| \| --- \| --- \| --- \| --- \| --- \| --- \| --- \| \| ◯ \| ◯ \| ◯ \| ◯ \| ◯ \| ◯ \| ◯ \|   **If you received these results, to what extent would you feel good about your treatment options for kidney cancer?**   \| Not at all 1 \| 2 \| 3 \| 4 \| 5 \| 6 \| Extremely 7 \| \| --- \| --- \| --- \| --- \| --- \| --- \| --- \| \| ‍ \|  \|  \|  \|  \|  \|  \| \| ◯  [PAGE BREAK] \| ◯ \| ◯ \| ◯ \| ◯ \| ◯ \| ◯ \| |
| Pilot, 1, 2 | **Please indicate to what extent you agree or disagree with each of the following statements:**   \|  \| Strongly disagree \| Disagree \| Somewhat disagree \| Neither agree nor disagree \| Somewhat agree \| Agree \| Strongly agree \| \| --- \| --- \| --- \| --- \| --- \| --- \| --- \| --- \| \| I trust healthcare professionals in the UK \| ◯ \| ◯ \| ◯ \| ◯ \| ◯ \| ◯ \| ◯ \| \| I trust the NHS \| ◯ \| ◯ \| ◯ \| ◯ \| ◯ \| ◯ \| ◯ \| \| I trust universities in the UK \| ◯ \| ◯ \| ◯ \| ◯ \| ◯ \| ◯ \| ◯ \| \| I trust the University of Cambridge \| ◯ \| ◯ \| ◯ \| ◯ \| ◯ \| ◯ \| ◯ \| \| I trust scientists \| ◯ \| ◯ \| ◯ \| ◯ \| ◯ \| ◯ \| ◯ \| \| I trust scientific knowledge \| ◯ \| ◯ \| ◯ \| ◯ \| ◯ \| ◯ \| ◯ \| \| I trust doctors \| ◯ \| ◯ \| ◯ \| ◯ \| ◯ \| ◯ \| ◯ \| \| I trust my own doctor \| ◯ \| ◯ \| ◯ \| ◯ \| ◯ \| ◯ \| ◯ \| |
| 1, 2 | \| I trust the pharmaceutical industry \| ◯ \| ◯ \| ◯ \| ◯ \| ◯ \| ◯ \| ◯ \| \| --- \| --- \| --- \| --- \| --- \| --- \| --- \| --- \| \| I trust GlaxoSmithKline \| ◯ \| ◯ \| ◯ \| ◯ \| ◯ \| ◯ \| ◯ \| \| [PAGE BREAK] \| \|  \|  \|  \|  \|  \|  \| |
| Pilot, 1, 2 | **How has the COVID-19 pandemic changed the amount of trust you have in the following:**   \|  \| Trust much less than before \| Trust less than before \| Trust somewhat less than before \| Trust the same amount as before \| Trust somewhat more than before \| Trust more than before \| Trust much more than before \| \| --- \| --- \| --- \| --- \| --- \| --- \| --- \| --- \| \| Healthcare professionals in the UK \| ◯ \| ◯ \| ◯ \| ◯ \| ◯ \| ◯ \| ◯ \| \| The NHS \| ◯ \| ◯ \| ◯ \| ◯ \| ◯ \| ◯ \| ◯ \| \| Universities in the UK \| ◯ \| ◯ \| ◯ \| ◯ \| ◯ \| ◯ \| ◯ \| \| The University of Cambridge \| ◯ \| ◯ \| ◯ \| ◯ \| ◯ \| ◯ \| ◯ \| \| Scientists \| ◯ \| ◯ \| ◯ \| ◯ \| ◯ \| ◯ \| ◯ \| \| Scientific knowledge \| ◯ \| ◯ \| ◯ \| ◯ \| ◯ \| ◯ \| ◯ \| \| Doctors \| ◯ \| ◯ \| ◯ \| ◯ \| ◯ \| ◯ \| ◯ \| \| My own doctor \| ◯ \| ◯ \| ◯ \| ◯ \| ◯ \| ◯ \| ◯ \| |
| 1, 2 | \| The pharmaceutical industry \| ◯ \| ◯ \| ◯ \| ◯ \| ◯ \| ◯ \| ◯ \| \| --- \| --- \| --- \| --- \| --- \| --- \| --- \| --- \| \| GlaxoSmithKline \| ◯ \| ◯ \| ◯ \| ◯ \| ◯ \| ◯ \| ◯ \| \| [PAGE BREAK] \| \|  \|  \|  \|  \|  \|  \| |
| Pilot, 1, 2 | **After they have all the information they need about their illness and possible treatments, some patients prefer to leave decisions about their treatment up to their doctor, while others prefer to participate in these decisions. Please check the statement that best describes what you believe would be ideal:**  ◯ The doctor should make the decisions using all that's known about the treatments.  ◯ The doctor should make the decisions but strongly consider the patient's opinion.  ◯ The doctor and the patient should make the decisions together on an equal basis.  ◯ The patient should make the decisions, but strongly consider the doctor's opinion.  ◯ The patient should make the decisions using all they know or learn about the treatments.  [PAGE BREAK] |
| 1, 2 | **How much do you associate the University of Cambridge with…**   \|  \| Not at all 1 \| 2 \| 3 \| 4 \| 5 \| 6 \| Very much 7 \| \| --- \| --- \| --- \| --- \| --- \| --- \| --- \| --- \| \| …Science? \| ◯ \| ◯ \| ◯ \| ◯ \| ◯ \| ◯ \| ◯ \| \| …Teaching? \| ◯ \| ◯ \| ◯ \| ◯ \| ◯ \| ◯ \| ◯ \| \| …Healthcare? \| ◯ \| ◯ \| ◯ \| ◯ \| ◯ \| ◯ \| ◯ \|   **How much do you associate the NHS with…**   \|  \| Not at all 1 \| 2 \| 3 \| 4 \| 5 \| 6 \| Very much 7 \| \| --- \| --- \| --- \| --- \| --- \| --- \| --- \| --- \| \| …Science? \| ◯ \| ◯ \| ◯ \| ◯ \| ◯ \| ◯ \| ◯ \| \| …Teaching? \| ◯ \| ◯ \| ◯ \| ◯ \| ◯ \| ◯ \| ◯ \| \| …Healthcare? \| ◯ \| ◯ \| ◯ \| ◯ \| ◯ \| ◯ \| ◯ \|   **How much do you associate GlaxoSmithKline with…**   \|  \| Not at all 1 \| 2 \| 3 \| 4 \| 5 \| 6 \| Very much 7 \| \| --- \| --- \| --- \| --- \| --- \| --- \| --- \| --- \| \| …Science? \| ◯ \| ◯ \| ◯ \| ◯ \| ◯ \| ◯ \| ◯ \| \| …Teaching? \| ◯ \| ◯ \| ◯ \| ◯ \| ◯ \| ◯ \| ◯ \| \| …Healthcare? \| ◯ \| ◯ \| ◯ \| ◯ \| ◯ \| ◯ \| ◯ \| \| ‍ \|  \|  \|  \|  \|  \|  \|  \| \| [PAGE BREAK] \| \|  \|  \|  \|  \|  \|  \| |
| 1, 2 | **How familiar are you with the University of Cambridge?**   \| Not at all 1 \| 2 \| 3 \| 4 \| 5 \| 6 \| Very much 7 \| \| --- \| --- \| --- \| --- \| --- \| --- \| --- \| \| ◯ \| ◯ \| ◯ \| ◯ \| ◯ \| ◯ \| ◯ \|   **How familiar are you with the NHS?**   \| Not at all 1 \| 2 \| 3 \| 4 \| 5 \| 6 \| Very much 7 \| \| --- \| --- \| --- \| --- \| --- \| --- \| --- \| \| ◯ \| ◯ \| ◯ \| ◯ \| ◯ \| ◯ \| ◯ \|   **How familiar are you with GlaxoSmithKline?**   \| Not at all 1 \| 2 \| 3 \| 4 \| 5 \| 6 \| Very much 7 \| \| --- \| --- \| --- \| --- \| --- \| --- \| --- \| \| ◯  [PAGE BREAK] \| ◯ \| ◯ \| ◯ \| ◯ \| ◯ \| ◯ \| |
| Pilot, 1, 2 | **How often are you online?**  ◯ Daily  ◯ Several times a week  ◯ Occasionally (a few times a month)  ◯ Rarely (a few times per year)  ◯ Never |
| Pilot, 1, 2 | **How confident are you in using online tools and information on the internet?**   \| Not at all confident 1 \| 2 \| 3 \| 4 \| 5 \| 6 \| Very confident 7 \| \| --- \| --- \| --- \| --- \| --- \| --- \| --- \| \| ◯  [PAGE BREAK] \| ◯ \| ◯ \| ◯ \| ◯ \| ◯ \| ◯ \| |
| Pilot, 1, 2 | **Almost done!**  **We will present a couple of puzzles that vary in difficulty. Please take your time and try to answer as many as you can. These help us understand how you think about numbers.**  [NOTE: The following questions comprise the Adaptive Berlin Numeracy test and were administered in the original adaptive format, i.e., Q2b only administered if Q1 was answered correctly, etc. Each is asked on a new page See Cokely et al. (2012) for details.]  [Q1]  **Out of 1,000 people in a small town 500 are members of a choir. Out of these 500 members in the choir 100 are men.  Out of the 500 inhabitants that are not in the choir 300 are men. What is the probability that a randomly drawn man is a member of the choir?**   Please indicate the probability in percent. Don't include the percentage sign (%).  ____  [Q2a]  **Imagine we are throwing a five-sided die 50 times. On average, out of these 50 throws how many times would this five-sided die show an odd number (1, 3 or 5)?**    ____ out of 50 throws.  [Q2b]  **Imagine we are throwing a loaded die (6 sides). The probability that the die shows a 6 is twice as high as the probability of each of the other numbers. On average, out of these 70 throws how  many times would the die show the number 6?**   ____  out of 70 throws.  [Q3]  **In a forest 20% of mushrooms are red, 50% brown and 30% white. A red mushroom is poisonous with a probability of 20%. A mushroom that is not red is poisonous with a probability of 5%. What is the probability that a poisonous mushroom in the forest is red?**  Please indicate the probability in percent. Don't include the percentage sign (%).  ____ |
| 1, 2 | **Which of the following numbers represents the biggest risk of getting a disease?**  ◯ 1 in 100  ◯ 1 in 1000  ◯ 1 in 10  **Imagine that we flip a fair coin 1,000 times. What is your best guess about how many times the coin would come up heads in 1,000 flips?**     ____ times out of 1,000  **In a scratch card lottery, the chance of winning a £10 prize on the card is 1%.**  **What is your best guess about how many people would win a £10 prize if 1,000 people each buy a single scratch card?**  ____ person(s) out of 1,000  **At a raffle, the chance of winning a car is 1 in 1,000.**    What percentage of tickets in the raffle win a car?    ____%. |
| Pilot, 1, 2 | **Information about you**  If you would prefer not to answer a question please leave it blank.  **What is your age?**  _____  **What is your gender?**  ◯ Female  ◯ Male  ◯ Other / prefer to self-describe (please specify below)  ____________________ ◯ Prefer not to say  [PAGE BREAK]  **Do you yourself have any direct experience with cancer?**  ◯ Yes, I personally have experience with cancer  ◯ Yes, someone close to me has experience with cancer  ◯ No  ◯ Prefer not to say  **Do you yourself have any direct experience with kidney cancer?**  ◯ Yes, I personally have experience with kidney cancer  ◯ Yes, someone close to me has experience with kidney cancer  ◯ No  ◯ Prefer not to say  [PAGE BREAK]  **Have you had any experience with any PREDICT tool before you participated in this research?**  ◯ Yes  ◯ No  ◯ Prefer not to say  [PAGE BREAK]  **Please indicate your highest educational qualification:**  ◯ No qualification  ◯ Primary school  ◯ GCSE / O-Level / BTEC NVQ Level 2  ◯ A-Level / International Baccalaureate / BTEC NVQ Level 3 |
| Pilot | ◯ Higher Education / Higher National Certificates and Diplomas |
| 1, 2 | ◯ Higher National Certificates and Diplomas / Other vocational |
| Pilot, 1, 2 | ◯ Bachelor’s degree or equivalent  ◯ Master’s degree / Postgraduate qualification  ◯ Doctoral degree  ◯ Prefer not to say  [PAGE BREAK]  **What is your native language?**  ◯ English  ◯ Other (please specify below)  ____________________  ◯ Prefer not to say  **If English is not your native language, what is your level of English?**  ◯ Beginner  ◯ Intermediate  ◯ Advanced  [PAGE BREAK]  **Ethnic background**  ◯ White  ◯ Mixed/Multiple ethnic groups  ◯ Asian/Asian British  ◯ Black/African/Caribbean/Black British  ◯ Other ethnic group (please specify below)  ____________________  ◯ Prefer not to say  [PAGE BREAK]  **Were you born in the UK?**  ◯ Yes  ◯ No  ◯ Prefer not to say  **How many years have you been living in the UK?**  If you prefer not to answer this question you may leave it blank.  ________ |

**Table S3.** Correlations among perceived certainty, accuracy, reliability and trustworthiness, and among trust in the tool as a whole, the selected treatment options, the numbers, and the algorithm.

*Pilot*

|  | Certain. | Accur. | Reliabil. | Trustw. | Trust in tool | Trust in treat. | Trust in num. | Trust in algo. | Weight |
| --- | --- | --- | --- | --- | --- | --- | --- | --- | --- |
| Certainty | - |  |  |  |  |  |  |  |  |
| Accuracy | 0.79 | - |  |  |  |  |  |  |  |
| Reliability | 0.77 | 0.77 | - |  |  |  |  |  |  |
| Trustworthiness | 0.71 | 0.72 | 0.81 | - |  |  |  |  |  |
| Trust in tool | 0.83 | 0.81 | 0.87 | 0.85 | - |  |  |  |  |
| Trust in treatments | 0.78 | 0.78 | 0.78 | 0.77 | 0.76 | - |  |  |  |
| Trust in numbers | 0.85 | 0.84 | 0.87 | 0.84 | 0.84 | 0.72 | - |  |  |
| Trust in algorithm | 0.82 | 0.83 | 0.84 | 0.82 | 0.80 | 0.66 | 0.84 | - |  |
| Weight on Predict | 0.26 | 0.25 | 0.28 | 0.27 | 0.31 | 0.18 | 0.29 | 0.27 | - |
| % weight on numbers | 0.12 | 0.11 | 0.14 | 0.11 | 0.13 | 0.10 | 0.16 | 0.09 | 0.16 |
| % weight on inst. | -0.11 | -0.09 | -0.11 | -0.07 | -0.07 | -0.06 | -0.12 | -0.13 | -0.01 |
| % weight on algorithm | -0.03 | -0.03 | -0.02 | -0.04 | -0.06 | -0.08 | -0.06 | 0.07 | -0.01 |
| % weight on data | 0.03 | 0.05 | 0.00 | 0.04 | 0.01 | 0.09 | 0.01 | 0.02 | -0.09 |
| % weight on other | -0.07 | -0.08 | -0.06 | -0.09 | -0.08 | -0.13 | -0.05 | -0.05 | -0.12 |
| Gen. inst. trust | 0.23 | 0.23 | 0.25 | 0.27 | 0.28 | 0.26 | 0.25 | 0.21 | 0.09 |
| Trust in cued inst. | 0.23 | 0.26 | 0.27 | 0.28 | 0.30 | 0.25 | 0.26 | 0.25 | 0.07 |

|  | % wt on num. | % wt on inst. | % wt on algo. | % wt on data | % wt on other | Gen. inst. tr. | Trust in cued inst. |
| --- | --- | --- | --- | --- | --- | --- | --- |
| Certainty |  |  |  |  |  |  |  |
| Accuracy |  |  |  |  |  |  |  |
| Reliability |  |  |  |  |  |  |  |
| Trustworthiness |  |  |  |  |  |  |  |
| Trust in tool |  |  |  |  |  |  |  |
| Trust in treatments |  |  |  |  |  |  |  |
| Trust in numbers |  |  |  |  |  |  |  |
| Trust in algorithm |  |  |  |  |  |  |  |
| Weight on Predict |  |  |  |  |  |  |  |
| % weight on numbers | - |  |  |  |  |  |  |
| % weight on inst. | -0.41 | - |  |  |  |  |  |
| % weight on algorithm | -0.38 | -0.14 | - |  |  |  |  |
| % weight on data | -0.41 | -0.31 | 0.04 | - |  |  |  |
| % weight on other | -0.22 | -0.17 | -0.16 | -0.21 | - |  |  |
| Gen. inst. trust | -0.00 | 0.09 | -0.06 | -0.04 | -0.01 | - |  |
| Trust in cued inst. | -0.07 | 0.17 | -0.05 | -0.05 | -0.03 | 0.73 | - |

*Experiment 1*

|  | Certain. | Accur. | Reliabil. | Trustw. | Trust in tool | Trust in treat. | Trust in num. | Trust in algo. | Weight |
| --- | --- | --- | --- | --- | --- | --- | --- | --- | --- |
| Certainty | - |  |  |  |  |  |  |  |  |
| Accuracy | 0.77 | - |  |  |  |  |  |  |  |
| Reliability | 0.81 | 0.79 | - |  |  |  |  |  |  |
| Trustworthiness | 0.76 | 0.76 | 0.85 | - |  |  |  |  |  |
| Trust in tool | 0.84 | 0.83 | 0.88 | 0.87 | - |  |  |  |  |
| Trust in treatments | 0.82 | 0.78 | 0.82 | 0.81 | 0.80 | - |  |  |  |
| Trust in numbers | 0.84 | 0.85 | 0.87 | 0.85 | 0.81 | 0.73 | - |  |  |
| Trust in algorithm | 0.83 | 0.83 | 0.88 | 0.85 | 0.81 | 0.72 | 0.83 | - |  |
| Weight on Predict | 0.23 | 0.27 | 0.28 | 0.27 | 0.29 | 0.20 | 0.29 | 0.27 | - |
| % weight on numbers | 0.11 | 0.12 | 0.12 | 0.13 | 0.13 | 0.10 | 0.17 | 0.08 | 0.19 |
| % weight on inst. | -0.09 | -0.11 | -0.13 | -0.09 | -0.10 | -0.07 | -0.13 | -0.12 | -0.05 |
| % weight on algorithm | -0.01 | 0.01 | 0.03 | 0.00 | -0.01 | -0.03 | -0.03 | 0.08 | 0.01 |
| % weight on data | 0.02 | 0.01 | 0.01 | 0.00 | 0.00 | 0.04 | -0.02 | 0.02 | -0.11 |
| % weight on other | -0.08 | -0.09 | -0.08 | -0.10 | -0.09 | -0.11 | -0.08 | -0.07 | -0.12 |
| Hypotheticality | -0.41 | -0.43 | -0.41 | -0.40 | -0.42 | -0.43 | -0.39 | -0.41 | -0.14 |
| Gen. inst. trust | 0.22 | 0.25 | 0.27 | 0.30 | 0.28 | 0.26 | 0.25 | 0.26 | 0.09 |
| Trust in cued inst. | 0.18 | 0.19 | 0.23 | 0.27 | 0.24 | 0.21 | 0.19 | 0.23 | 0.12 |
| Familiarity of cued inst. | 0.03 | 0.04 | 0.04 | 0.07 | 0.08 | 0.04 | 0.04 | 0.03 | 0.04 |

|  | % wt on num. | % wt on inst. | % wt on algo. | % wt on data | % wt on other | Hypotheticality | Gen. inst. tr. | Trust in cued inst. | Fam of cued inst. |
| --- | --- | --- | --- | --- | --- | --- | --- | --- | --- |
| Certainty |  |  |  |  |  |  |  |  |  |
| Accuracy |  |  |  |  |  |  |  |  |  |
| Reliability |  |  |  |  |  |  |  |  |  |
| Trustworthiness |  |  |  |  |  |  |  |  |  |
| Trust in tool |  |  |  |  |  |  |  |  |  |
| Trust in treatments |  |  |  |  |  |  |  |  |  |
| Trust in numbers |  |  |  |  |  |  |  |  |  |
| Trust in algorithm |  |  |  |  |  |  |  |  |  |
| Weight on Predict |  |  |  |  |  |  |  |  |  |
| % weight on numbers | - |  |  |  |  |  |  |  |  |
| % weight on inst. | -0.42 | - |  |  |  |  |  |  |  |
| % weight on algorithm | -0.44 | -0.08 | - |  |  |  |  |  |  |
| % weight on data | -0.44 | -0.30 | 0.09 | - |  |  |  |  |  |
| % weight on other | -0.24 | -0.13 | -0.13 | -0.22 | - |  |  |  |  |
| Hypotheticality | -0.08 | 0.07 | 0.01 | -0.03 | 0.09 | - |  |  |  |
| Gen. inst. trust | -0.01 | 0.08 | -0.02 | 0.00 | -0.07 | -0.17 | - |  |  |
| Trust in cued inst. | -0.07 | 0.27 | -0.02 | -0.12 | -0.08 | -0.14 | 0.62 | - |  |
| Familiarity of cued inst. | -0.07 | 0.24 | -0.06 | -0.12 | 0.00 | -0.06 | 0.17 | 0.44 | - |

*Experiment 2*

|  | Certain. | Accur. | Reliabil. | Trustw. | Trust in tool | Trust in treat. | Trust in num. | Trust in algo. | Weight |
| --- | --- | --- | --- | --- | --- | --- | --- | --- | --- |
| Certainty | - |  |  |  |  |  |  |  |  |
| Accuracy | 0.80 | - |  |  |  |  |  |  |  |
| Reliability | 0.77 | 0.73 | - |  |  |  |  |  |  |
| Trustworthiness | 0.75 | 0.72 | 0.83 | - |  |  |  |  |  |
| Trust in tool | 0.81 | 0.78 | 0.83 | 0.82 | - |  |  |  |  |
| Trust in treatments | 0.78 | 0.74 | 0.77 | 0.76 | 0.73 | - |  |  |  |
| Trust in numbers | 0.81 | 0.80 | 0.82 | 0.82 | 0.70 | 0.64 | - |  |  |
| Trust in algorithm | 0.80 | 0.80 | 0.81 | 0.81 | 0.73 | 0.61 | 0.76 | - |  |
| Weight on Predict | 0.19 | 0.20 | 0.22 | 0.23 | 0.22 | 0.17 | 0.22 | 0.21 | - |
| % weight on numbers | 0.09 | 0.09 | 0.12 | 0.12 | 0.11 | 0.08 | 0.16 | 0.06 | 0.10 |
| % weight on inst. | -0.11 | -0.11 | -0.12 | -0.10 | -0.09 | -0.07 | -0.13 | -0.13 | -0.05 |
| % weight on algorithm | -0.03 | -0.01 | -0.03 | -0.02 | -0.02 | -0.07 | -0.08 | 0.10 | 0.00 |
| % weight on data | 0.04 | 0.03 | 0.02 | 0.01 | 0.01 | 0.04 | 0.02 | 0.02 | -0.04 |
| % weight on other | -0.03 | -0.03 | -0.04 | -0.06 | -0.04 | -0.03 | -0.04 | -0.04 | -0.04 |
| Hypotheticality | -0.32 | -0.35 | -0.29 | -0.29 | -0.31 | -0.32 | -0.29 | -0.28 | -0.07 |
| Gen. inst. trust | 0.27 | 0.25 | 0.30 | 0.30 | 0.29 | 0.27 | 0.25 | 0.27 | 0.13 |
| Trust in cued inst. | 0.21 | 0.20 | 0.24 | 0.27 | 0.24 | 0.22 | 0.21 | 0.23 | 0.12 |
| Familiarity of cued inst. | 0.13 | 0.13 | 0.14 | 0.14 | 0.14 | 0.13 | 0.13 | 0.13 | 0.05 |

|  | % wt on num. | % wt on inst. | % wt on algo. | % wt on data | % wt on other | Hypotheticality | Gen. inst. tr. | Trust in cued inst. | Fam of cued inst. |
| --- | --- | --- | --- | --- | --- | --- | --- | --- | --- |
| Certainty |  |  |  |  |  |  |  |  |  |
| Accuracy |  |  |  |  |  |  |  |  |  |
| Reliability |  |  |  |  |  |  |  |  |  |
| Trustworthiness |  |  |  |  |  |  |  |  |  |
| Trust in tool |  |  |  |  |  |  |  |  |  |
| Trust in treatments |  |  |  |  |  |  |  |  |  |
| Trust in numbers |  |  |  |  |  |  |  |  |  |
| Trust in algorithm |  |  |  |  |  |  |  |  |  |
| Weight on Predict |  |  |  |  |  |  |  |  |  |
| % weight on numbers | - |  |  |  |  |  |  |  |  |
| % weight on inst. | -0.39 | - |  |  |  |  |  |  |  |
| % weight on algorithm | -0.40 | -0.11 | - |  |  |  |  |  |  |
| % weight on data | -0.39 | -0.32 | -0.04 | - |  |  |  |  |  |
| % weight on other | -0.21 | -0.14 | -0.15 | -0.25 | - |  |  |  |  |
| Hypotheticality | -0.06 | 0.04 | 0.05 | 0.01 | -0.00 | - |  |  |  |
| Gen. inst. trust | 0.01 | 0.09 | -0.05 | -0.00 | -0.08 | -0.17 | - |  |  |
| Trust in cued inst. | -0.06 | 0.21 | -0.08 | -0.07 | -0.02 | -0.13 | 0.61 | - |  |
| Familiarity of cued inst. | -0.08 | 0.17 | -0.05 | -0.07 | 0.04 | -0.09 | 0.17 | 0.44 | - |

**Table S4.** Confirmatory factor analyses for four-factor models vs. one-factor models.

|  | Four-factor model | | | | | One-factor model | | | | | Chi-squared difference test | |
| --- | --- | --- | --- | --- | --- | --- | --- | --- | --- | --- | --- | --- |
|  | AIC | BIC | RMSEA | CFI | TLI | AIC | BIC | RMSEA | CFI | TLI | X^2^ diff | *p* |
| Pilot: certainty, accuracy, reliability, trustworthiness | 33939 | 34121 | .150 | .851 | .818 | 34932 | 35085 | .179 | .774 | .739 | 1005 | <.001 |
| Pilot: trust in tool, treatments, numbers, algorithm | 34170 | 34352 | .159 | .833 | .796 | As above | | | | | 774 | <.001 |
| Experiment 1: certainty, accuracy, reliability, trustworthiness | 67282 | 67490 | .165 | .837 | .800 | 68926 | 69101 | .186 | .780 | .746 | 1656 | <.001 |
| Experiment 1: trust in tool, treatments, numbers, algorithm | 66913 | 67121 | .158 | .850 | .816 | As above | | | | | 2025 | <.001 |
| Experiment 2: certainty, accuracy, reliability, trustworthiness | 85610 | 85824 | .179 | .771 | .720 | 86695 | 86875 | .188 | .733 | .691 | 1097 | <.001 |
| Experiment 2: trust in tool, treatments, numbers, algorithm | 83265 | 83479 | .144 | .853 | .820 | As above | | | | | 3442 | <.001 |

**Table S5.** Coefficients and *p*-values for covariates in linear regression models, pilot experiment. Each model included uncertainty cue, institutional cue, generalized institutional trust, and trust in the cued institution. Because trust in the cued institution was included, these models necessarily only included participants who were shown an institution cue (*n* = 645). Generalized institutional trust and trust in cued institution were correlated at *r* = 0.73.

|  |  | Generalized institutional trust | | Trust in cued institution | |
| --- | --- | --- | --- | --- | --- |
|  | Adjusted *r^2^* | *B* | *p* | *B* | *p* |
| **Primary measures** |  |  |  |  |  |
| Trustworthiness | 0.092 | 0.29 | <.001*** | 0.13 | 0.03* |
| Certainty | 0.063 | 0.21 | 0.007** | 0.13 | 0.03* |
| Accuracy | 0.075 | 0.19 | 0.004** | 0.12 | 0.02* |
| Reliability | 0.083 | 0.25 | 0.001** | 0.13 | 0.02* |
| **Exploratory measures** |  |  |  |  |  |
| Trust in tool as a whole | 0.106 | 0.28 | <.001*** | 0.13 | 0.02* |
| Trust in treatments | 0.071 | 0.22 | 0.003** | 0.12 | 0.03* |
| Trust in numbers | 0.081 | 0.27 | <.001*** | 0.11 | 0.05* |
| Trust in algorithm | 0.067 | 0.17 | 0.02* | 0.15 | 0.008** |
| Weight placed on tool | 0.011 | 4.21 | 0.03* | -0.96 | 0.50 |
| % wt on numbers | 0.004 | 2.19 | 0.13 | -2.34 | 0.03* |
| % wt on institutions | 0.045 | -1.02 | 0.41 | 3.01 | 0.001** |
| % wt on algorithm | 0.002 | 0.09 | 0.91 | -0.49 | 0.42 |
| % wt on data | 0.006 | -1.86 | 0.09 | 0.35 | 0.67 |
| % wt on other | 0.002 | 0.59 | 0.54 | -0.54 | 0.46 |
| Worry | 0.053 | -0.23 | <.001*** | -0.00 | 0.99 |
| Information sufficiency | 0.030 | 0.33 | 0.01* | 0.09 | 0.35 |
| Decision confidence | 0.027 | 0.22 | <.001*** | -0.01 | 0.80 |
| Change in decision confidence | -0.004 | -0.02 | 0.80 | 0.03 | 0.50 |

**Table S6.** Coefficients and *p*-values for covariates in linear regression models, Experiment 1. Each model included uncertainty cue, institutional cue, generalized institutional trust, and trust in the cued institution. Because trust in the cued institution was included, these models necessarily only included participants who were shown an institution cue (*n* = 1329). Generalized institutional trust and trust in cued institution were correlated at *r* = 0.62.

|  |  | Generalized institutional trust | | Trust in cued institution | |
| --- | --- | --- | --- | --- | --- |
|  | Adjusted *r^2^* | *B* | *p* | *B* | *p* |
| **Primary measures** |  |  |  |  |  |
| Trustworthiness | 0.102 | 0.28 | <.001*** | 0.12 | <.001*** |
| Certainty | 0.052 | 0.22 | <.001*** | 0.05 | 0.08 |
| Accuracy | 0.064 | 0.23 | <.001*** | 0.05 | 0.11 |
| Reliability | 0.082 | 0.25 | <.001*** | 0.09 | 0.005** |
| **Exploratory measures** |  |  |  |  |  |
| Trust in tool as a whole | 0.087 | 0.24 | <.001*** | 0.08 | 0.009** |
| Trust in treatments | 0.075 | 0.25 | <.001*** | 0.06 | 0.06 |
| Trust in numbers | 0.061 | 0.24 | <.001*** | 0.07 | 0.03* |
| Trust in algorithm | 0.078 | 0.23 | <.001*** | 0.10 | 0.002** |
| Weight placed on tool | 0.013 | 0.50 | 0.62 | 1.77 | 0.01* |
| % wt on numbers | 0.006 | 1.39 | 0.10 | -1.26 | 0.03* |
| % wt on institutions | 0.102 | -0.93 | 0.15 | 2.56 | <.001*** |
| % wt on algorithm | 0.008 | -0.38 | 0.38 | 0.15 | 0.62 |
| % wt on data | 0.026 | 1.11 | 0.08 | -1.26 | 0.004** |
| % wt on other | 0.009 | -1.18 | 0.01* | -0.20 | 0.56 |
| Worry | 0.037 | -0.15 | <.001*** | -0.07 | 0.02* |
| Information sufficiency | 0.042 | 0.24 | <.001*** | 0.11 | 0.04* |
| Decision confidence | 0.024 | 0.11 | 0.004** | 0.05 | 0.07 |
| Change in decision confidence | 0.003 | -0.02 | 0.67 | 0.05 | 0.04* |

**Table S7.** Coefficients and *p*-values for covariates in linear regression models, Experiment 2. Each model included uncertainty cue, institutional cue, generalized institutional trust, and trust in the cued institution. Because trust in the cued institution was included, these models necessarily only included participants who were shown an institution cue (*n* = 1551). Generalized institutional trust and trust in cued institution were correlated at *r* = 0.61.

|  |  | Generalized institutional trust | | Trust in cued institution | |
| --- | --- | --- | --- | --- | --- |
|  | Adjusted *r^2^* | *B* | *p* | *B* | *p* |
| **Primary measures** |  |  |  |  |  |
| Trustworthiness | 0.118 | 0.41 | <.001*** | 0.08 | 0.02* |
| Certainty | 0.089 | 0.37 | <.001*** | 0.03 | 0.38 |
| Accuracy | 0.081 | 0.32 | <.001*** | 0.03 | 0.29 |
| Reliability | 0.109 | 0.40 | <.001*** | 0.05 | 0.09 |
| **Exploratory measures** |  |  |  |  |  |
| Trust in tool as a whole | 0.112 | 0.39 | <.001*** | 0.04 | 0.22 |
| Trust in treatments | 0.090 | 0.35 | <.001*** | 0.03 | 0.25 |
| Trust in numbers | 0.080 | 0.36 | <.001*** | 0.07 | 0.04* |
| Trust in algorithm | 0.097 | 0.39 | <.001*** | 0.04 | 0.17 |
| Weight placed on tool | 0.016 | 1.70 | 0.11 | 1.94 | 0.008** |
| % wt on numbers | 0.006 | 1.40 | 0.07 | -1.43 | 0.006** |
| % wt on institutions | 0.046 | -0.93 | 0.14 | 2.81 | <.001*** |
| % wt on algorithm | 0.004 | -0.34 | 0.47 | -0.38 | 0.23 |
| % wt on data | 0.005 | 1.14 | 0.07 | -1.25 | 0.004** |
| % wt on other | 0.006 | -1.27 | 0.004** | 0.25 | 0.41 |
| Worry | 0.025 | -0.21 | <.001*** | -0.02 | 0.61 |
| Information sufficiency | 0.041 | 0.42 | <.001*** | 0.04 | 0.44 |
| Decision confidence | 0.040 | 0.25 | <.001*** | -0.01 | 0.81 |
| Change in decision confidence | 0.009 | 0.07 | 0.05* | 0.01 | 0.71 |

**Table S8**

Coefficients and *p*-values for covariates in logistic regression models, pilot experiment. Each model included uncertainty cue, institutional cue, generalized institutional trust, and trust in the cued institution, and necessarily only included participants who were shown an institution cue.

|  | *Model with inst., unc. cues only* | *Model with institutional cue, uncertainty cue, generalized institutional trust, and trust in cued institution* | | | | |
| --- | --- | --- | --- | --- | --- | --- |
|  |  |  | *Generalized institutional trust* | | *Trust in cued institution* | |
| **Dependent variable** | *McFadden pseudo r^2^* | *McFadden*  *pseudo r^2^* | *B* | *p* | *B* | *p* |
| *Switched their choice after viewing tool  (Switched = 1)*  *n = 645* | 0.004 | 0.011 | -0.06 | 0.76 | 0.19 | 0.18 |
| *Switched to active treatment after viewing tool (Switched = 1) (Only those who initially chose passive treatment)*  *n = 209* | 0.007 | 0.036 | 0.48 | 0.12 | -0.01 | 0.96 |
| *Switched to passive treatment after viewing tool (Switched = 1)*  *(Only those who initially chose active treatment)*  *n = 436* | 0.003 | 0.012 | -0.31 | 0.21 | 0.26 | 0.15 |

**Table S9**

Coefficients and *p*-values for covariates in logistic regression models, Experiment 1. Each model included uncertainty cue, institutional cue, generalized institutional trust, and trust in the cued institution, and necessarily only included participants who were shown an institution cue.

|  | *Model with inst., unc. cues only* | *Model with institutional cue, uncertainty cue, generalized institutional trust, and trust in cued institution* | | | | |
| --- | --- | --- | --- | --- | --- | --- |
|  |  |  | *Generalized institutional trust* | | *Trust in cued institution* | |
| **Dependent variable** | *McFadden pseudo r^2^* | *McFadden*  *pseudo r^2^* | *B* | *p* | *B* | *p* |
| *Switched their choice after viewing tool  (Switched = 1)*  *n = 1329* | 0.005 | 0.008 | -0.01 | 0.96 | 0.10 | 0.19 |
| *Switched to active treatment after viewing tool (Switched = 1) (Only those who initially chose passive treatment)*  *n = 377* | 0.009 | 0.056 | 0.67 | <.001*** | -0.08 | 0.52 |
| *Switched to passive treatment after viewing tool (Switched = 1)*  *(Only those who initially chose active treatment)*  *n = 952* | 0.005 | 0.010 | -0.26 | 0.08 | 0.18 | 0.09 |

**Table S10**

Coefficients and *p*-values for covariates in logistic regression models, Experiment 2. Each model included uncertainty cue, institutional cue, generalized institutional trust, and trust in the cued institution, and necessarily only included participants who were shown an institution cue.

|  | *Model with inst., unc. cues only* | *Model with institutional cue, uncertainty cue, generalized institutional trust, and trust in cued institution* | | | | |
| --- | --- | --- | --- | --- | --- | --- |
|  |  |  | *Generalized institutional trust* | | *Trust in cued institution* | |
| **Dependent variable** | *McFadden pseudo r^2^* | *McFadden*  *pseudo r^2^* | *B* | *p* | *B* | *p* |
| *Switched their choice after viewing tool  (Switched = 1)*  *n = 1551* | 0.003 | 0.005 | -0.12 | 0.30 | 0.14 | 0.06 |
| *Switched to active treatment after viewing tool (Switched = 1) (Only those who initially chose passive treatment)*  *n = 518* | 0.006 | 0.027 | 0.16 | 0.32 | 0.21 | 0.06 |
| *Switched to passive treatment after viewing tool (Switched = 1)*  *(Only those who initially chose active treatment)*  *n = 1033* | 0.004 | 0.006 | -0.17 | 0.35 | 0.06 | 0.63 |

**Table S11**

Mean values and standard deviations across all conditions.

|  |  | Mean (Standard Deviation) | | |
| --- | --- | --- | --- | --- |
|  |  | Pilot | Experiment 1 | Experiment 2 |
| **Dependent variables (possible range)** |  |  |  |  |
| Trustworthiness (1-7) |  | 5.06 (1.13) | 5.05 (1.16) | 4.98 (1.12) |
| Certainty (1-7) |  | 5.14 (1.04) | 5.06 (1.05) | 4.98 (1.02) |
| Accuracy (1-7) |  | 5.09 (0.94) | 5.05 (0.97) | 5.01 (0.95) |
| Reliability (1-7) |  | 5.06 (1.06) | 5.03 (1.10) | 4.99 (1.06) |
| Trust in tool as a whole (1-7) |  | 5.06 (1.01) | 5.08 (1.02) | 5.03 (1.00) |
| Trust in treatments (1-7) |  | 5.35 (1.00) | 5.31 (1.02) | 5.24 (0.99) |
| Trust in numbers (1-7) |  | 5.02 (1.07) | 4.93 (1.14) | 4.83 (1.19) |
| Trust in algorithm (1-7) |  | 4.92 (1.08) | 4.87 (1.11) | 4.86 (1.10) |
| Weight placed on tool (0-100) |  | 68.01 (24.94) | 69.27 (23.75) | 67.19 (23.96) |
| % wt on numbers (0-100) |  | 32.84 (19.31) | 34.07 (19.85) | 31.53 (17.17) |
| % wt on institutions (0-100) |  | 22.85 (16.02) | 20.93 (15.05) | 20.02 (13.85) |
| % wt on algorithm (0-100) |  | 16.49 (10.89) | 16.26 (10.10) | 18.49 (10.42) |
| % wt on data (0-100) |  | 23.68 (14.94) | 24.93 (14.77) | 25.94 (14.14) |
| % wt on other (0-100) |  | 4.14 (11.78) | 3.81 (11.28) | 4.02 (10.53) |
| Worry (1-5 Exp 1; 1-7 Exp 2 & 3) |  | 2.96 (0.74) | 3.98 (1.03) | 3.93 (1.06) |
| Information sufficiency (1-7) |  | 4.51 (1.74) | 4.47 (1.72) | 4.42 (1.72) |
| Decision confidence (1-5) |  | 3.90 (0.87) | 3.86 (0.90) | 3.80 (0.90) |
| Change in decision confidence |  | +0.42 (0.81) | +0.32 (0.81) | +0.36 (0.80) |
| **Covariates (possible range)** |  |  |  |  |
| Generalized institutional trust (1-7) |  | 5.74 (0.80) | 5.35 (0.88) | 5.44 (0.76) |
| Trust in cued institution (1-7) |  | 5.72 (1.05) | 5.09 (1.48) | 5.04 (1.33) |
| Familiarity with cued institution (1-7) |  | - | 4.59 (1.98) | 3.50 (1.88) |
| Hypotheticality (1-7) |  | - | 2.63 (0.99) | 2.71 (0.96) |

**Table S12**

Preregistered and exploratory ANOVAs, Experiment 2.

|  | Uncertainty cues | | | Institutional cues | | | Uncertainty cue × institutional cue interaction | | |
| --- | --- | --- | --- | --- | --- | --- | --- | --- | --- |
|  | *F* | *p* | *η^2^_G_* | *F* | *p* | *η^2^_G_* | *F* | *p* | *η^2^_G_* |
| **Preregistered ANOVAs** |  |  |  |  |  |  |  |  |  |
| Trustworthiness | 2.77 | 0.06 | 0.003 | 2.51 | 0.06 | 0.004 | 0.64 | 0.70 | 0.002 |
| Trust in tool as a whole | 3.14 | 0.04* | 0.003 | 2.20 | 0.09 | 0.003 | 0.68 | 0.67 | 0.002 |
| **Exploratory ANOVAs** |  |  |  |  |  |  |  |  |  |
| Certainty | 1.86 | 0.16 | 0.002 | 1.75 | 0.16 | 0.003 | 0.82 | 0.55 | 0.002 |
| Accuracy | 3.24 | 0.04* | 0.003 | 1.22 | 0.30 | 0.002 | 0.45 | 0.85 | 0.001 |
| Reliability | 3.24 | 0.04* | 0.003 | 2.34 | 0.07 | 0.003 | 0.73 | 0.62 | 0.002 |
| Trust in treatments | 2.26 | 0.10 | 0.002 | 1.44 | 0.23 | 0.002 | 1.08 | 0.37 | 0.003 |
| Trust in numbers | 3.02 | 0.05* | 0.003 | 1.06 | 0.36 | 0.002 | 0.48 | 0.82 | 0.001 |
| Trust in algorithm | 1.83 | 0.16 | 0.002 | 2.00 | 0.11 | 0.003 | 0.72 | 0.64 | 0.002 |
| Weight placed on tool | 0.12 | 0.89 | 0.000 | 0.32 | 0.81 | 0.000 | 1.11 | 0.35 | 0.003 |
| % wt on numbers | 2.69 | 0.07 | 0.003 | 2.25 | 0.08 | 0.003 | 0.96 | 0.45 | 0.003 |
| % wt on institutions | 1.11 | 0.33 | 0.001 | 20.73 | <.001*** | 0.029 | 0.94 | 0.47 | 0.003 |
| % wt on algorithm | 0.30 | 0.74 | 0.000 | 1.90 | 0.13 | 0.003 | 1.86 | 0.08 | 0.005 |
| % wt on data | 0.50 | 0.61 | 0.000 | 2.20 | 0.09 | 0.003 | 0.87 | 0.51 | 0.003 |
| % wt on other | 2.54 | 0.08 | 0.002 | 1.85 | 0.14 | 0.003 | 0.71 | 0.64 | 0.002 |
| Worry | 0.60 | 0.55 | 0.001 | 0.36 | 0.78 | 0.001 | 1.05 | 0.39 | 0.003 |
| Information sufficiency | 1.82 | 0.16 | 0.002 | 0.74 | 0.53 | 0.001 | 0.54 | 0.78 | 0.002 |
| Decision confidence | 1.86 | 0.16 | 0.002 | 0.45 | 0.72 | 0.001 | 0.17 | 0.98 | 0.000 |
| Change in decision confidence | 5.11 | 0.006** | 0.005 | 2.62 | 0.05* | 0.004 | 1.44 | 0.20 | 0.004 |

***Note.*** * = p < .05; ** = p < .01; *** = p < .001.

**Table S13**

Coefficients and *p*-values for covariates in linear regression models across all participants (n = 2,068), Experiment 2. Each model included uncertainty cue, institutional cue, and a single covariate.

| Model | *Base model (all participants)* | *Base model + generalized institutional trust (all participants)* | | | *Base model + hypotheticality (all participants)* | | |
| --- | --- | --- | --- | --- | --- | --- | --- |
|  | *Model* | *Generalized institutional trust* | | *Model* | *Hypotheticality* | | *Model* |
|  | *Adjusted r^2^* | *B* | *p* | *Adjusted r^2^* | *B* | *p* | *Adjusted r^2^* |
| **Primary measures** |  |  |  |  |  |  |  |
| Trustworthiness | .004 | .44 | <.001*** | .092 | -.33 | <.001*** | .085 |
| Certainty | .002 | .36 | <.001*** | .074 | -.34 | <.001*** | .103 |
| Accuracy | .003 | .31 | <.001*** | .063 | -.35 | <.001*** | .127 |
| Reliability | .004 | .41 | <.001*** | .090 | -.32 | <.001*** | .088 |
| **Exploratory measures** |  |  |  |  |  |  |  |
| Trust in tool as a whole | .004 | .38 | <.001*** | .088 | -.33 | <.001*** | .102 |
| Trust in treatments | .002 | .35 | <.001*** | .074 | -.33 | <.001*** | .104 |
| Trust in numbers | .002 | .38 | <.001*** | .062 | -.36 | <.001*** | .086 |
| Trust in algorithm | .002 | .39 | <.001*** | .076 | -.32 | <.001*** | .082 |
| Weight placed on tool | -.002 | 4.05 | <.001*** | .014 | -1.64 | .003** | .002 |
| % wt on numbers | .003 | .24 | .63 | .003 | -1.15 | .003** | .007 |
| % wt on institutions | .028 | 1.52 | <.001*** | .034 | .56 | .07 | .029 |
| % wt on algorithm | .001 | -.67 | .03* | .003 | .51 | .03* | .002 |
| % wt on data | .001 | -.03 | .94 | .001 | .09 | .79 | .001 |
| % wt on other | .003 | -1.06 | <.001*** | .008 | -.00 | .99 | .002 |
| Worry | -.001 | -.23 | <.001*** | .025 | .12 | <.001*** | .010 |
| Information sufficiency | .000 | .42 | <.001*** | .034 | -.29 | <.001*** | .026 |
| Decision confidence | .000 | .22 | <.001*** | .034 | -.14 | <.001*** | .022 |
| Change in decision confidence | .006 | .08 | <.001*** | .011 | -.05 | .005** | .010 |

**Table S14**

Coefficients and *p*-values for covariates in linear regression models across participants shown institutional cues (n = 1,551), Experiment 2. Each model included uncertainty cue, institutional cue, and a single covariate.

| Model | *Base model (participants shown institutional cues only)* | *Base model + trust in cued institution (participants shown institutional cues only)* | | | *Base model + familiarity with cued institution (participants shown institutional cues only)* | | | *Base model + generalized institutional trust (participants shown institutional cues only)* | | |
| --- | --- | --- | --- | --- | --- | --- | --- | --- | --- | --- |
|  | *Model* | *Trust in cued institution* | | *Model* | *Familiarity with cued institution* | | *Model* | *Generalized institutional trust* | | *Model* |
|  | *Adjusted r^2^* | *B* | *p* | *Adjusted r^2^* | *B* | *p* | *Adjusted r^2^* | *B* | *p* | *Adjusted r^2^* |
| **Primary measures** |  |  |  |  |  |  |  |  |  |  |
| Trustworthiness | .004 | .27 | <.001*** | .077 | .08 | <.001*** | .018 | .49 | <.001*** | .116 |
| Certainty | -.000 | .20 | <.001*** | .048 | .08 | <.001*** | .016 | .40 | <.001*** | .089 |
| Accuracy | .002 | .18 | <.001*** | .046 | .07 | <.001*** | .018 | .35 | <.001*** | .081 |
| Reliability | .003 | .23 | <.001*** | .066 | .08 | <.001*** | .020 | .45 | <.001*** | .108 |
| **Exploratory measures** |  |  |  |  |  |  |  |  |  |  |
| Trust in tool as a whole | .003 | .22 | <.001*** | .063 | .08 | <.001*** | .019 | .43 | <.001*** | .111 |
| Trust in treatments | .001 | .19 | <.001*** | .051 | .07 | <.001*** | .016 | .38 | <.001*** | .090 |
| Trust in numbers | .001 | .24 | <.001*** | .052 | .08 | <.001*** | .015 | .43 | <.001*** | .078 |
| Trust in algorithm | .003 | .23 | <.001*** | .057 | .08 | <.001*** | .016 | .44 | <.001*** | .096 |
| Weight placed on tool | -.001 | 2.73 | <.001*** | .016 | .68 | .05 | .001 | 3.66 | <.001*** | .013 |
| % wt on numbers | .002 | -.78 | .04* | .004 | -.72 | .004** | .007 | -.05 | .93 | .002 |
| % wt on institutions | .010 | 2.38 | <.001*** | .045 | 1.18 | <.001*** | .030 | 1.92 | <.001*** | .020 |
| % wt on algorithm | .001 | -.53 | .02* | .004 | -.15 | .32 | .001 | -.72 | .03* | .003 |
| % wt on data | .001 | -.72 | .02* | .004 | -.46 | .03* | .003 | -.12 | .79 | .000 |
| % wt on other | -.000 | -.34 | .12 | .001 | .15 | .30 | -.000 | -1.02 | .002** | .006 |
| Worry | -.001 | -.11 | <.001*** | .013 | -.02 | .22 | -.001 | -.23 | <.001*** | .025 |
| Information sufficiency | -.001 | .23 | <.001*** | .023 | .05 | .04* | .002 | .46 | <.001*** | .041 |
| Decision confidence | -.001 | .11 | <.001*** | .017 | .04 | .001** | .005 | .24 | <.001*** | .041 |
| Change in decision confidence | .004 | .04 | .02* | .007 | -.02 | .09 | .006 | .08 | .002** | .010 |

**Table S15**
Coefficients and *p*-values for covariates in logistic regression models including uncertainty cue, institutional cue, and a single covariate for exploratory binary measures, Experiment 2. McFadden’s pseudo-R^2^s (43) are simply one minus the ratio of the model’s log-likelihood to that of a model with intercept only, and reflect the variance accounted for by the logistic regression model.

|  | Model | | | | | | | | | |
| --- | --- | --- | --- | --- | --- | --- | --- | --- | --- | --- |
|  | Inst., unc. cues only | | Inst., unc. cues + generalized  institutional trust | | | | Inst., unc. cues + hypotheticality | | | |
|  |  |  |  |  | *Generalized institutional trust* | |  |  | *Hypotheticality* | |
| **Dependent variable** | *pseudo r^2^* | | *pseudo r^2^* | | *B* | *p* | *pseudo r^2^* | | *B* | *p* |
| Switched their choice after viewing tool  (Switched = 1)  N = 2068 | .003 | | .003 | | .09 | .24 | .003 | | -.06 | .30 |
| Switched to active treatment after viewing tool (Switched = 1) (Only those who initially chose passive treatment: N = 689) | .006 | | .023 | | .40 | <.001*** | .006 | | .03 | .74 |
| Switched to passive treatment after viewing tool (Switched = 1)  (Only those who initially chose active treatment: N = 1379) | .004 | | .004 | | -.04 | .76 | .007 | | -.15 | .10 |

**Table S16**

Coefficients and *p*-values for covariates in logistic regression models including uncertainty cue, institutional cue, and a single covariate for exploratory binary measures, restricted to participants who were shown an institutional cue, Experiment 2. McFadden’s pseudo-R^2^s (43) are simply one minus the ratio of the model’s log-likelihood to that of a model with intercept only, and reflect the variance accounted for by the logistic regression model.

|  | Model | | | | | | | | | | | | |
| --- | --- | --- | --- | --- | --- | --- | --- | --- | --- | --- | --- | --- | --- |
|  | Inst., unc. cues only | | Inst., unc. cues + trust in cued institution | | | Inst., unc. cues + familiarity with cued institution | | | | Inst., unc. cues + generalized institutional trust | | | |
|  |  |  |  |  | *Trust in cued institution* |  |  | *Familiarity with*  *cued institution* | |  |  |  | *Generalized institutional trust* |
| **Dependent variable** | *pseudo r^2^* | | *pseudo r^2^* | *B* | *p* |  | *pseudo r^2^* | *B* | *p* |  | *pseudo r^2^* | *B* | *p* |
| Switched their choice after viewing tool  (Switched = 1)  (Only those who were shown an institutional cue: N = 1551) | .003 | | .005 | .09 | .11 | .005 | | .06 | .08 |  | .003 | .03 | 0.74 |
| Switched to active treatment after viewing tool (Switched = 1) (Only those who initially chose passive treatment and were shown an institutional cue: N = 518) | .006 | | .025 | 0.29 | <.001*** | .010 | | .08 | .10 |  | .022 | .37 | .002** |
| Switched to passive treatment after viewing tool (Switched = 1)  (Only those who initially chose active treatment and were shown an institutional cue: N = 1033) | .004 | | .004 | -.012 | .86 | .005 | | .05 | .44 |  | .005 | -.11 | .42 |

**Pilot - Additional Results**

In ANCOVAs, the same pattern was observed with respect to which main effects were significant and nonsignificant, with the exception that weak significant effects of institution cues on trustworthiness, trust in the tool as a whole, and trust in treatments emerged when generalized institutional trust was controlled for. The weak effects of institution cue on the weight placed on the tool, as well as the proportion of weight placed on the numbers, the algorithm, and the data became insignificant when trust in the cued institution was controlled for, although these analyses necessarily omitted participants who had not viewed an institution cue.

**Experiment 1 - Additional Results**

The weak main effects of institutional cues on trustworthiness and trust in the tool as a whole were not significant in ANCOVAs which controlled for generalized institutional trust; all other (non)significant effects in Table 6 remained (non)significant in these ANCOVAs. As in Experiment 1, the proportion of weight placed on institutions was greatest when the tool was labeled with the NHS’s logo, less when labeled with Cambridge’s logo, and least when labeled with no logo. Post-hoc analyses of the main effect on information sufficiency revealed that this variable was significantly higher for the group shown the Cambridge cue (mean 4.67, 95% CI 4.52–4.83) than the groups shown the GlaxoSmithKline cue (mean 4.31, 95% CI 4.15–4.47) or no cue at all (mean 4.37, 95% CI 4.21–4.52). Counterintuitively, Tukey’s post-hocs suggested that weight placed on data was significantly *higher* among participants shown the “less well trusted” cue (mean 25.8, 95% CI: 24.6–26.9) than among those shown no uncertainty cue (mean 23.7, 95% CI: 22.6-24.9), although there was substantial overlap in 95% confidence intervals. Participants shown the “less well trusted” cue were less apt to feel that they had received sufficient information (mean 4.35, 95% CI 4.21–4.49) than those shown no uncertainty information (mean 4.62, 95% CI 4.49–4.76).

The model using trust in cued institution as a covariate accounted for more variance (directionally) than the model using familiarity in the cued institution for all variables other than *proportion of weight on algorithm.* See Table S6 for analyses that include generalized institutional trust and trust in cued institution in the same model.

**Experiment 2 - Additional Results**

There was a main effect of institutional cue on the amount that decision confidence increased, with Tukey’s post-hocs suggesting a greater increase for the no-logo group (mean 0.42, 95% CI 0.35 – 0.48) than the University of Cambridge group (mean 0.29, 95% CI 0.22 – 0.35). There was also a main effect of uncertainty cue, with a greater confidence increase for “There are other factors which affect outcomes” (mean 0.42, 95% CI 0.36 – 0.48) than for “The tool’s estimates are less well tested…” (mean 0.29, 95% CI 0.23 – 0.35). In the case of the institutional cue effect, this may have been due to the chance difference in initial confidence scores (before participants were shown the stimulus) between the groups who would be shown the University of Cambridge cue (mean 3.5, 95% CI 3.4 – 3.6) vs. no cue (mean 3.3, 95% CI 3.3 – 3.4). No such differences in initial confidence scores existed between the groups shown different uncertainty cues.

In ANCOVAs that controlled for generalized institutional trust, the only main effects that remained significant were the main effect of uncertainty cue on accuracy, the main effect of institutional cue on the proportion of weight placed on institutions, and the main effects of uncertainty and institutional cues on change in decision confidence. All effects that were nonsignificant in the ANOVAs remained nonsignificant in the ANCOVAs.

## References

1. Candido dos Reis FJ, Wishart GC, Dicks EM, Greenberg D, Rashbass J, Schmidt MK, et al. An updated PREDICT breast cancer prognostication and treatment benefit prediction model with independent validation. Breast Cancer Res. 2017;19(1):1–13.

2. Thurtle DR, Greenberg DC, Lee LS, Huang HH, Pharoah PD, Gnanapragasam VJ. Individual prognosis at diagnosis in nonmetastatic prostate cancer: Development and external validation of the PREDICT: Prostate multivariable model. PLoS Med. 2019;16(3):1–19.

3. Carver T, Hartley S, Lee A, Cunningham AP, Archer S, de Villiers CB, et al. Canrisk tool—A web interface for the prediction of breast and ovarian cancer risk and the likelihood of carrying genetic pathogenic variants. Cancer Epidemiol Biomarkers Prev. 2021;30(3):469–73.

4. Kanis JA, Hans D, Cooper C, Baim S, Bilezikian JP, Binkley N, et al. Interpretation and use of FRAX in clinical practice. Osteoporos Int. 2011;22(9):2395–411.

5. Lopez-Gonzalez AA, Aguilo A, Frontera M, Bennasar-Veny M, Campos I, Vicente-Herrero T, et al. Effectiveness of the Heart Age tool for improving modifiable cardiovascular risk factors in a Southern European population: A randomized trial. Eur J Prev Cardiol. 2015;22(3):389–96.

6. Hippisley-Cox J, Coupland C, Brindle P. Development and validation of QRISK3 risk prediction algorithms to estimate future risk of cardiovascular disease: prospective cohort study. BMJ [Internet]. 2017 May 23;(357):j2099. Available from: http://www.bmj.com/lookup/doi/10.1136/bmj.j2099

7. van der Bles AM, van der Linden S, Freeman ALJ, Mitchell J, Galvao AB, Zaval L, et al. Communicating uncertainty about facts, numbers and science. R Soc Open Sci. 2019 May 31;6(5).

8. Stewart KJ. Trust transfer on the World Wide Web. Organ Sci. 2003;14(1):5–17.

9. Cazier JA. A framework and model for understanding the creation and sources of trust. Assoc Inf Syst - 11th Am Conf Inf Syst AMCIS 2005 A Conf a Hum Scale. 2005;1(2):44–7.

10. Mayer et al. An Integrative Model of Organizational Trust Author ( s ): Roger C . Mayer , James H . Davis and F . David Schoorman Published by : Academy of Management Stable URL : http://www.jstor.com/stable/258792 REFERENCES Linked references are available on JSTOR f. Acad Manag Rev. 1995;20(3):709–34.

11. Trope Y, Liberman N. Construal Theory. Psychol Rev. 2010;117(2):440–63.

12. Fiske ST, Cuddy AJC, Glick P. Universal dimensions of social cognition: warmth and competence. Trends Cogn Sci. 2007;11(2):77–83.

13. Johnson BB, Slovic P. Presenting Uncertainty in Health Risk Assessment: Initial Studies of Its Effects on Risk Perception and Trust. Risk Anal [Internet]. 1995 Aug;15(4):485–94. Available from: http://doi.wiley.com/10.1111/j.1539-6924.1995.tb00341.x

14. Kreps SE, Kriner DL. Model uncertainty, political contestation, and public trust in science: Evidence from the COVID-19 pandemic. Sci Adv. 2020;6(43).

15. Lipkus IM, Klein WMP, Rimer BK. Communicating breast cancer risks to women using different formats. Cancer Epidemiol Biomarkers Prev. 2001;10(8):895–8.

16. Han PKJJ, Klein WMPP, Lehman T, Killam B, Massett H, Freedman AN. Communication of uncertainty regarding individualized cancer risk estimates: effects and influential factors. Med Decis Mak. 2011;31(2):354–66.

17. Johnson BB, Slovic P. Explaining Uncertainty in Health Risk Assessment: Effects on Risk Perception and Trust. Risk Anal [Internet]. 1995;15(4):1539–6924. Available from: https://illiad.mst.edu/illiad/illiad.dll?Action=10&Form=75&Value=244723

18. Longman T, Turner RM, King M, McCaffery KJ. The effects of communicating uncertainty in quantitative health risk estimates. Patient Educ Couns. 2012;89(2):252–9.

19. van der Bles AM, van der Linden S, Freeman ALJ, Spiegelhalter DJ. The effects of communicating uncertainty on public trust in facts and numbers. Proc Natl Acad Sci U S A. 2020;117(14):7672–83.

20. Schneider CR, Freeman ALJ, Spiegelhalter D, van der Linden S. The effects of communicating scientific uncertainty on trust and decision making in a public health context. Judgm Decis Mak. 2022;17(4):849–82.

21. Schneider CR, Freeman ALJ, Spiegelhalter D, van der Linden S. The effects of quality of evidence communication on perception of public health information about COVID-19: Two randomised controlled trials (Supplementary information). PLoS One [Internet]. 2021;16(11):e0259048. Available from: http://dx.doi.org/10.1371/journal.pone.0259048

22. Schneider CR, Freeman A, Spiegelhalter D, van der Linden S. The effects of communicating scientific uncertainty on trust and decision making in a public health context. Judgm Decis Mak. 2022;17(4):849–82.

23. Jiang P, Jones DB, Javie S. How Third-Party Certification Programs Relate to Consumer Trust in Online Transactions: An Exploratory Study. Psychol Mark [Internet]. 2008;25(9):839–58. Available from: http://eprints.lancs.ac.uk/23431/

24. van Baal S. Not all seals are equal: An experimental investigation of the effect of third-party seals on purchase probability in electronic commerce. Vol. 15, Electronic Commerce Research. 2015. 177–206 p.

25. Rafaeli A, Sagy Y, Derfler-Rozin R. Logos and initial compliance: A strong case of mindless trust. Organ Sci. 2008;19(6):845–59.

26. Lowry PB, Roberts TL, Higbee T. First Impressions with Websites: The Effect of the Familiarity and Credibility of Corporate Logos on Perceived Consumer Swift Trust of Websites. In: 12th International Conference on HCI. Beijing; 2007. p. 77–85.

27. Trachtenberg F, Dugan E, Hall MA. Practice recommendations How patients’ trust relates to their involvement in medical care ORIGINAL RESEARCH. J Fam Pract. 2005;54(4).

28. Cao J, Galinsky AD. The Diversity-Uncertainty-Valence (DUV) model of generalized trust development. Organ Behav Hum Decis Process [Internet]. 2020;161(March):49–64. Available from: https://doi.org/10.1016/j.obhdp.2020.03.007

29. Sønderskov KM, Daugbjerg C. Eco-Labelling, the State and Consumer Confidence. Polit Stud. 2010;(April):1–25.

30. Rothstein B. How is social capital produced? In: Social traps and the problem of trust. Cambridge: Cambridge University Press; 2005. p. 92–128.

31. Lee JD, See KA. Trust in automation: Designing for appropriate reliance. Hum Factors. 2004;46(1):50–80.

32. Zipf GK. Human behavior and the principle of least effort: An introduction to human ecology. Cambridge: Addison-Wesley; 1949.

33. Ahn J, Kim J, Sung Y. AI-powered recommendations: the roles of perceived similarity and psychological distance on persuasion. Int J Advert [Internet]. 2021;40(8):1366–84. Available from: https://doi.org/10.1080/02650487.2021.1982529

34. Thomsen M, Karsten S, Oort FJ. Distance in schools: the influence of psychological and structural distance from management on teachers’ trust in management, organisational commitment, and organisational citizenship behaviour. Sch Eff Sch Improv [Internet]. 2016;27(4):594–612. Available from: http://dx.doi.org/10.1080/09243453.2016.1158193

35. Lii YS, Chien CS, Pant A, Lee M. The challenges of long-distance relationships: The effects of psychological distance between service provider and consumer on the efforts to recover from service failure. J Appl Soc Psychol. 2013;43(6):1121–35.

36. Benedicktus RL. Psychological distance perceptions and trust beliefs for Internet-only and hybrid retailers: Implications for marketers. Diss Abstr Int Sect A Humanit Soc Sci [Internet]. 2009;69(7-A):2794. Available from: http://ezproxy.lib.cam.ac.uk:2048/login?url=http://search.ebscohost.com/login.aspx?direct=true&db=psyh&AN=2009-99010-402&site=ehost-live&scope=site

37. Cui Y, Mou J, Cohen J, Liu Y, Kurcz K. Understanding consumer intentions toward cross-border m-commerce usage: A psychological distance and commitment-trust perspective. Electron Commer Res Appl [Internet]. 2020;39(December 2019):100920. Available from: https://doi.org/10.1016/j.elerap.2019.100920

38. Fishbein M, Raven BH. The AB scales: An operational definition of belief and attitude. Hum Relations. 1962;15(1):35–44.

39. Farmer GD, Pearson M, Skylark WJ, Freeman ALJ, Spiegelhalter DJ. Redevelopment of the Predict: Breast Cancer website and recommendations for developing interfaces to support decision-making. Cancer Med. 2021;10(15):5141–53.

40. González S, Smith C. The Accuracy of Measures of Institutional Trust in Household Surveys: Evidence from the OECD Trust Database. OECD Stat Work Pap No 2017/11. 2017;1–39.

41. PytlikZillig LM, Kimbrough CD, Shockley E, Neal TMS, Herian MN, Hamm JA, et al. A longitudinal and experimental study of the impact of knowledge on the bases of institutional trust. Vol. 12, PLoS ONE. 2017. 1–31 p.

42. Kerr JR, Schneider CR, Freeman ALJ, Marteau T. Transparent communication of evidence does not undermine public trust in evidence. PNAS Nexus. 2022;(December):1–11.

43. McFadden D. Conditional logit analysis of qualitative choice behavior. In: Zarembka P, editor. Frontiers in Econometrics. Academic Press; 1974. p. 105–42.

44. Cokely ET, Galesic M, Schulz E, Ghazal S, Garcia-Retamero R. Measuring risk literacy: The Berlin Numeracy Test. Judgm Decis Mak. 2012;7(1):25–47.

45. Recchia G, van der Bles AM, Freeman ALJ. PREDICT: the potential pitfalls of visualisations of risk. Breast Cancer Res Treat (published Abstr [Internet]. 2020;180(2):527–596. Available from: https://link.springer.com/article/10.1007/s10549-019-05514-3#Sec243

1. https://www.prolific.co/ [↑](#footnote-ref-1)
